# Supplementary material for: ATP-Red enables single-cell bioenergetic phenotyping by flow cytometry
Source: Cell Rep Methods. 2026 Jul 30;6(9):101537. doi: 10.1016/j.crmeth.2026.101537 (PMC13615503; doi:10.1016/j.crmeth.2026.101537)
Supplement: Supplementary file 1 [file mmc1.pdf]

Cell Reports Methods, Volume *6*

Supplemental information

ATP-Red enables single-cell bioenergetic phenotyping by flow cytometry

Jose Marin, Bharati Singh, Amanda Fuchs, Tatiana Tarasenko, Emily Warren, Martha Kirby, Stacie Anderson, Stephen M. Wincovitch, Eliza M. Gordon-Lipkin, Shannon Kruk, A. Phillip West, and Peter J. McGuire

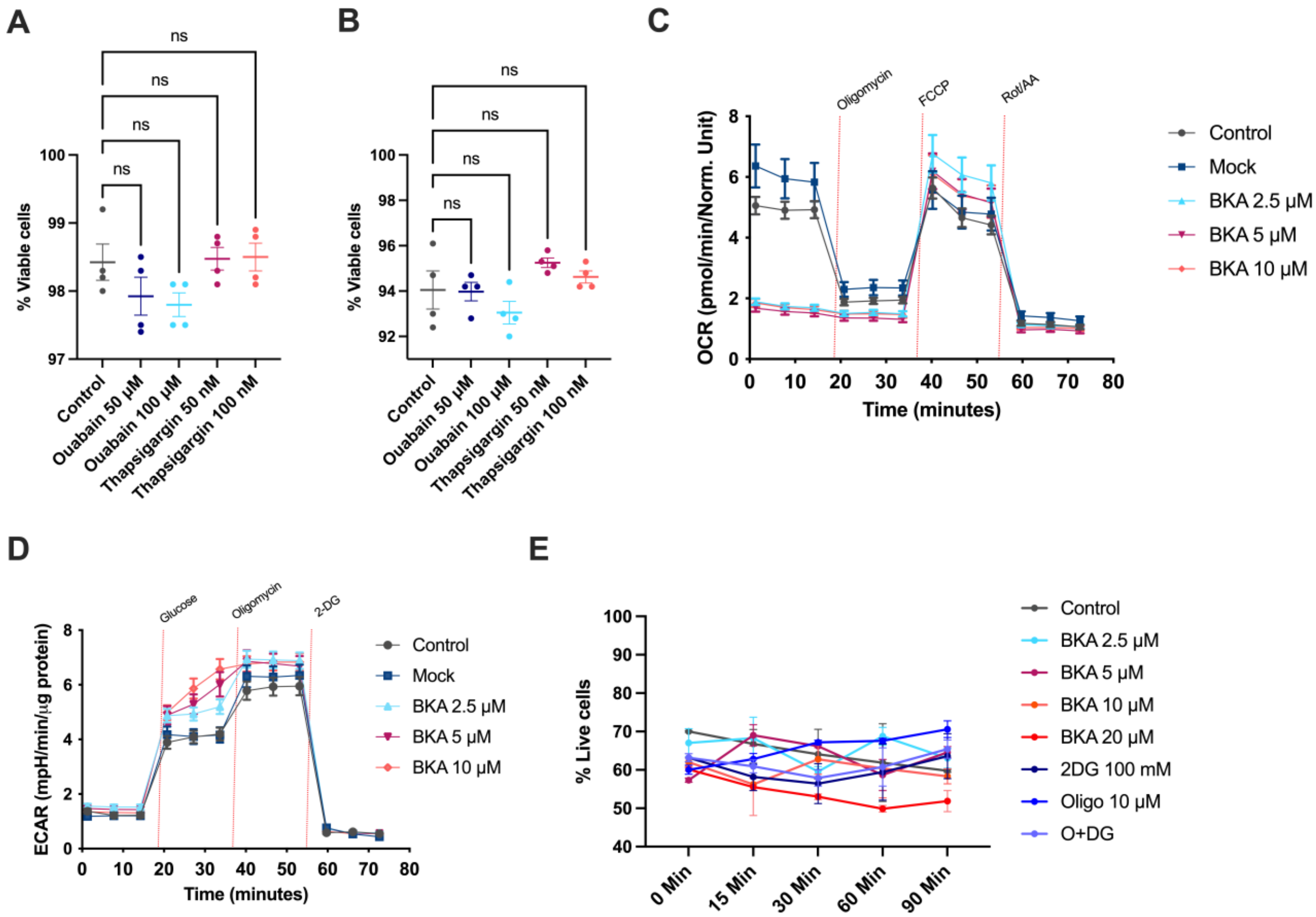

Figure S1. Cell viability and acute extracellular flux responses associated with ATPase inhibition, ANT inhibition, and short-term metabolic perturbation in MDCK cells. (A-B) Cell viability in MDCK cells after treatment with ouabain or thapsigargin under the conditions used for ATP accumulation experiments. Viability remained high across all conditions, with no significant reduction relative to control. (C) Representative OCR traces from Seahorse Mito Stress analysis of control and BKA-treated MDCK cells following sequential injection of oligomycin, FCCP, and rotenone/antimycin A. (D) Representative ECAR traces from Seahorse Glycolysis Stress analysis of control and BKA-treated MDCK cells following sequential injection of glucose, oligomycin, and 2-DG. (E) Cell viability during short term kinetic ATP-Red experiments in MDCK cells treated with BKA, 2-DG, oligomycin, or oligomycin plus 2-DG for the indicated times. Viability remained broadly stable during the 90 min acquisition period across all treatment conditions. Data are shown as mean ± SD. Statistical analysis for panels A-B was performed using ordinary one-way ANOVA followed by multiple-comparisons testing against control. Panel F was analyzed by ordinary two-way ANOVA with treatment and time as factors, followed by multiple-comparisons testing as indicated. Panels D-E are representative Seahorse traces. ns, not significant; *P < 0.05; **P < 0.01; ***P < 0.001; ****P < 0.0001.

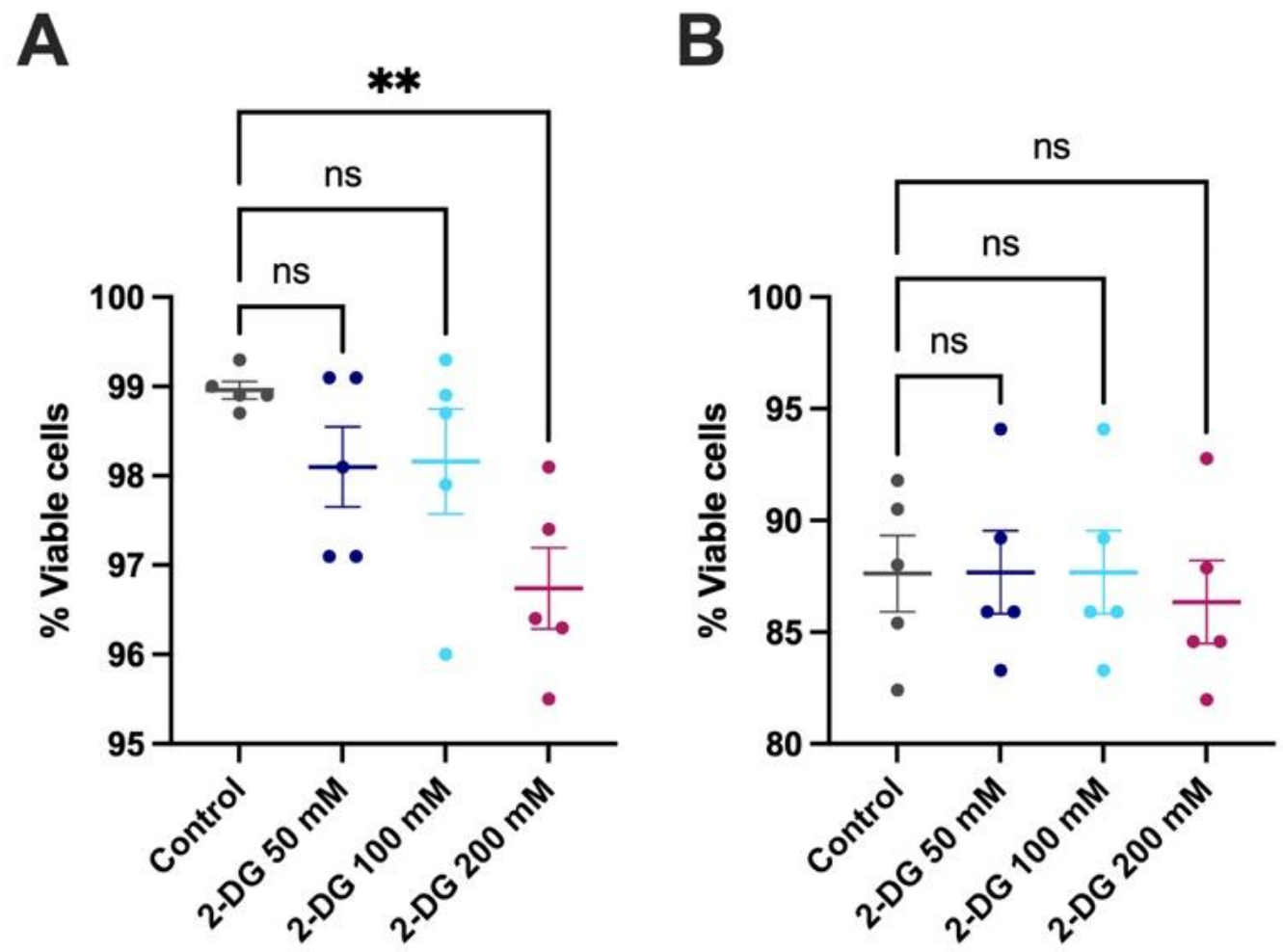

Figure S2. Cell viability remains preserved during glycolytic inhibition by 2-deoxy-D-glucose in MDCK cells. (A-B) Cell viability in MDCK cells treated with increasing concentrations of 2-deoxy-D-glucose (2-DG) under the conditions used for ATP/ADP, ATP-Red measurements. Viability remained high across all treatment conditions, with only modest changes at the highest dose. Data are shown as mean ± SD. Statistical analysis was performed using ordinary one-way ANOVA followed by multiple-comparisons testing against control. ns, not significant; *P < 0.05; **P < 0.01; ***P < 0.001; ****P < 0.0001.

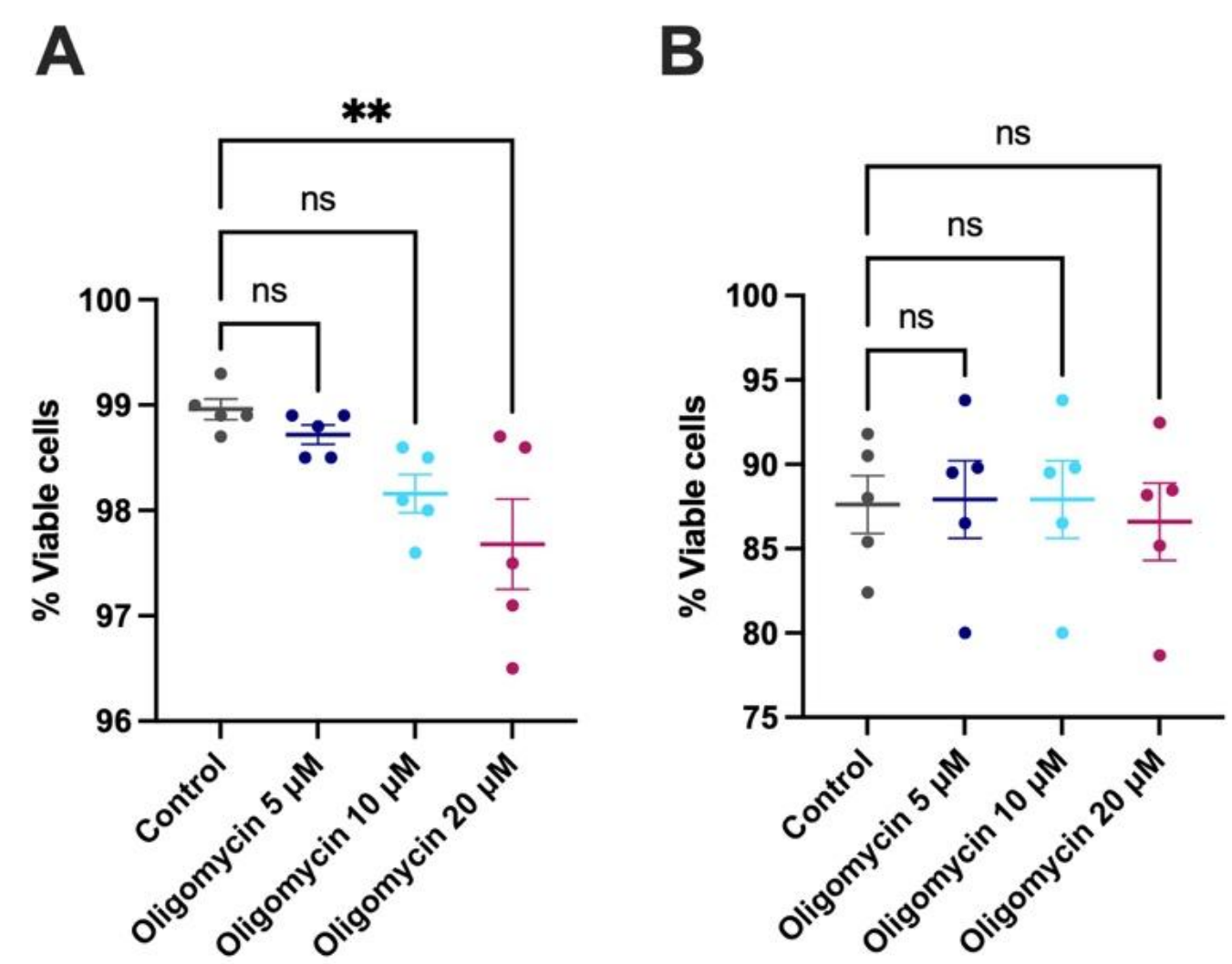

Figure S3. Cell viability remains largely preserved during mitochondrial ATP synthase inhibition by oligomycin in MDCK cells. (A-B) Cell viability in MDCK cells treated with increasing concentrations of oligomycin under the conditions used for ATP/ADP, ATP-Red measurements. Viability remained high across all treatment groups, with only limited reduction at the highest concentration. Data are shown as mean ± SD. Statistical analysis was performed using

ordinary one-way ANOVA followed by multiple-comparisons testing against control. ns, not significant; *P < 0.05; **P < 0.01; ***P < 0.001; ****P < 0.0001.

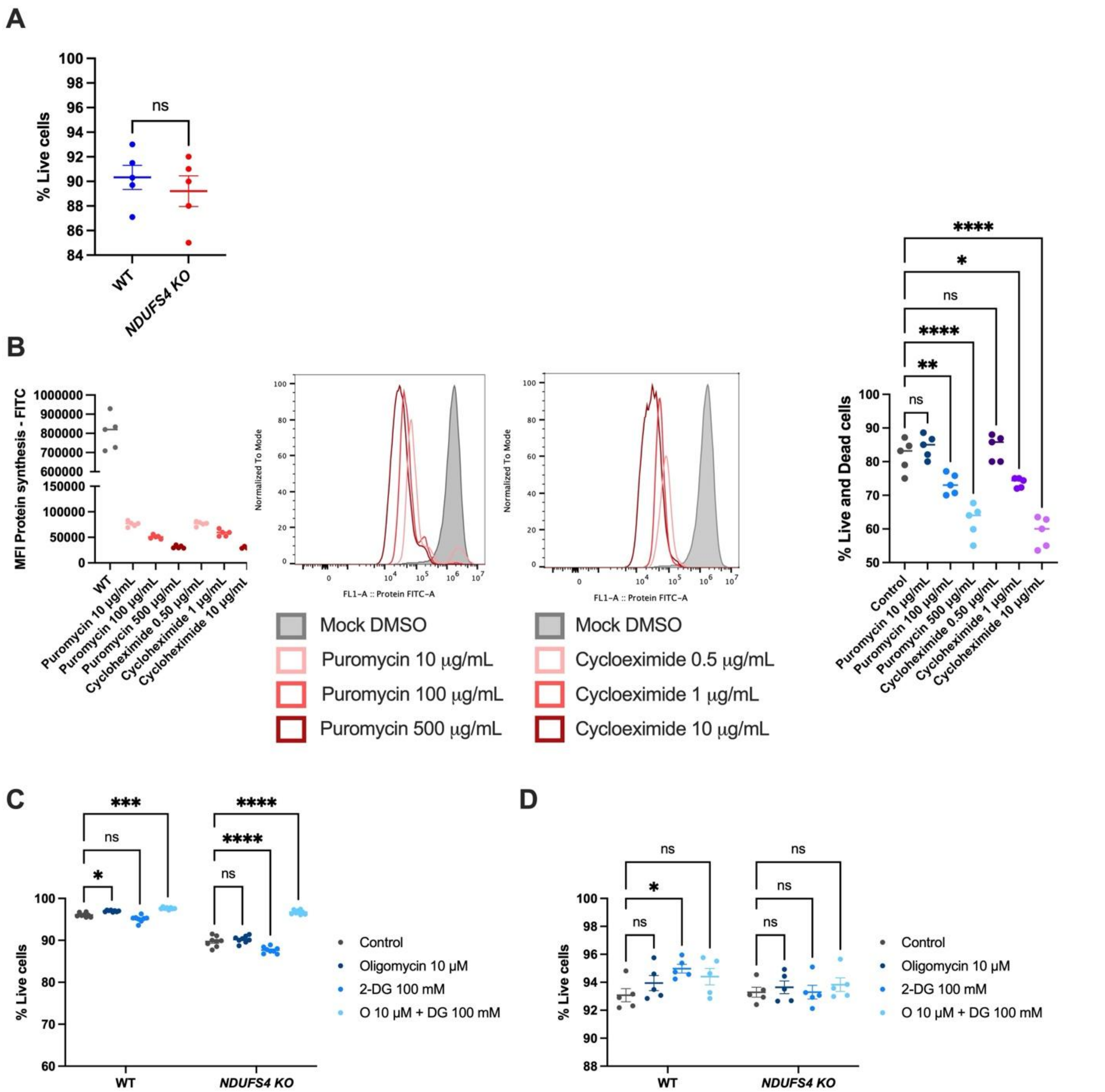

Figure S4. Viability controls and puromycin optimization for metabolic phenotyping in WT and *NDUFS4 KO* MDCK cells. (A) Baseline viability of WT and NDUFS4 KO MDCK cells. No significant difference in viability was observed between genotypes under untreated conditions. (B) Optimization of puromycin and cycloheximide treatment conditions for protein synthesis measurements in WT MDCK cells. Left, mean fluorescence intensity of protein synthesis signal across puromycin and cycloheximide conditions. Middle and right, representative histograms illustrating progressive reduction in protein synthesis signal with increasing puromycin or cycloheximide treatment relative to mock control. Far right, percentage of live and dead cells

across the indicated puromycin and cycloheximide conditions. (C-D) Viability of WT and *NDUFS4 KO* MDCK cells after treatment with oligomycin, 2-DG, or oligomycin plus 2-DG under the conditions used for ATP-Red-based metabolic dependence analysis. Viability remained broadly preserved across conditions, with only modest treatment-associated changes. Data are shown as mean ± SD. Statistical analysis for panel A was performed using unpaired two-tailed Student's t test. Panels B–D were analyzed using ordinary one-way ANOVA or two-way ANOVA, as appropriate for grouped comparisons, followed by multiple-comparisons testing as indicated on the graphs. Representative histograms are shown in panel B. ns, not significant; *P < 0.05; **P < 0.01; ***P < 0.001; ****P < 0.0001.

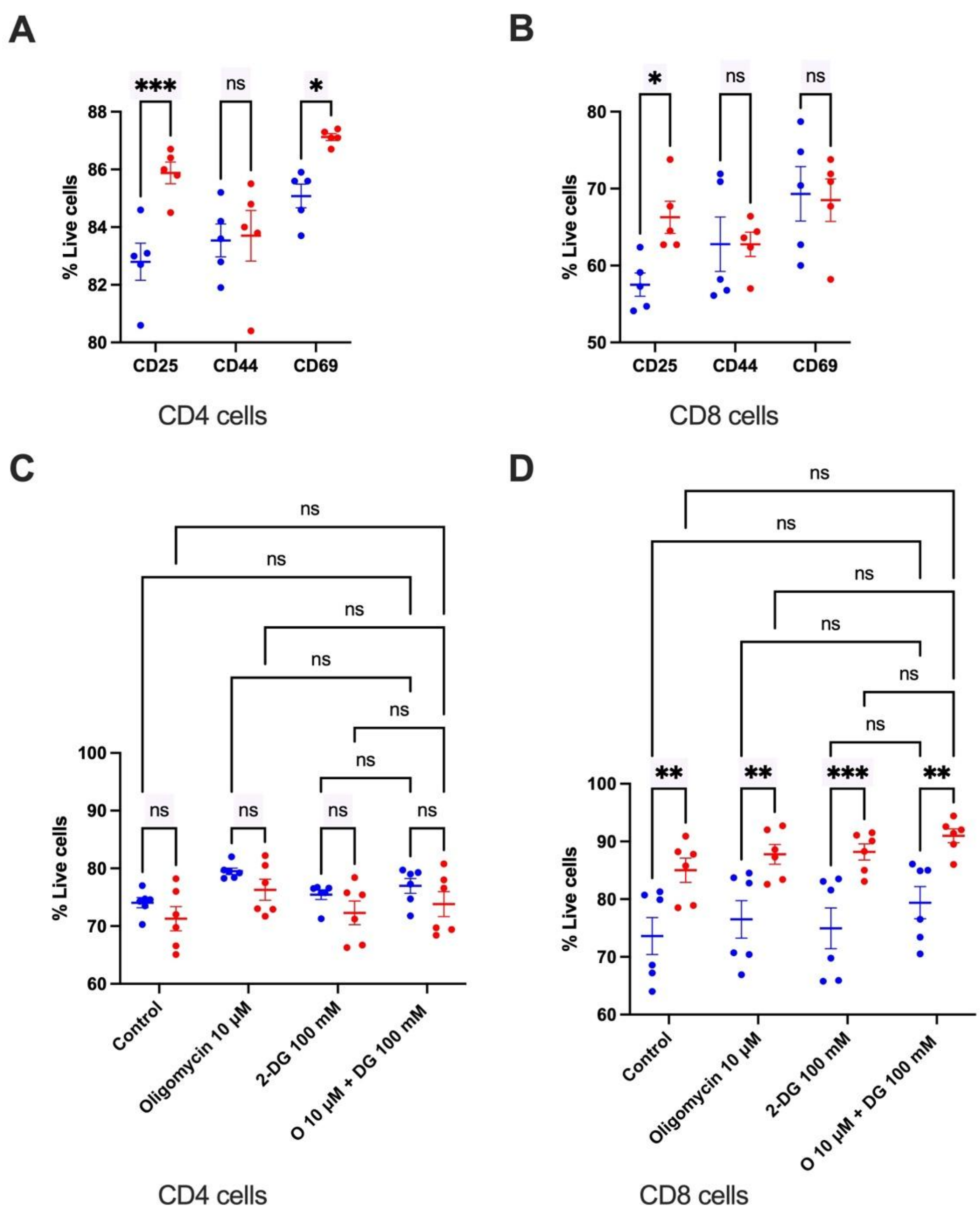

Figure S5. Viability controls for ATP-Red-based metabolic profiling during T cell activation. (A-B) Viability of activated-marker-positive $CD4^+$ and $CD8^+$ T cells after anti-CD3/CD28 stimulation. Activated cells expressing CD25, CD44, or CD69 maintained equal or greater viability than their resting counterparts. (C-D) Viability of $CD4^+$ and $CD8^+$ T cells under control conditions or after treatment with oligomycin, 2-DG, or oligomycin plus 2-DG during ATP-Red-based metabolic dependence analysis. Viability remained broadly preserved across all treatment groups, including under dual inhibition, with $CD8^+$ T cells showing robust survival. Data are shown as mean ± SD. Statistical analysis was performed using ordinary two-way ANOVA followed by multiple-comparisons testing as indicated. ns, not significant; *$P < 0.05$; **$P < 0.01$; ***$P < 0.001$; ****$P < 0.0001$.

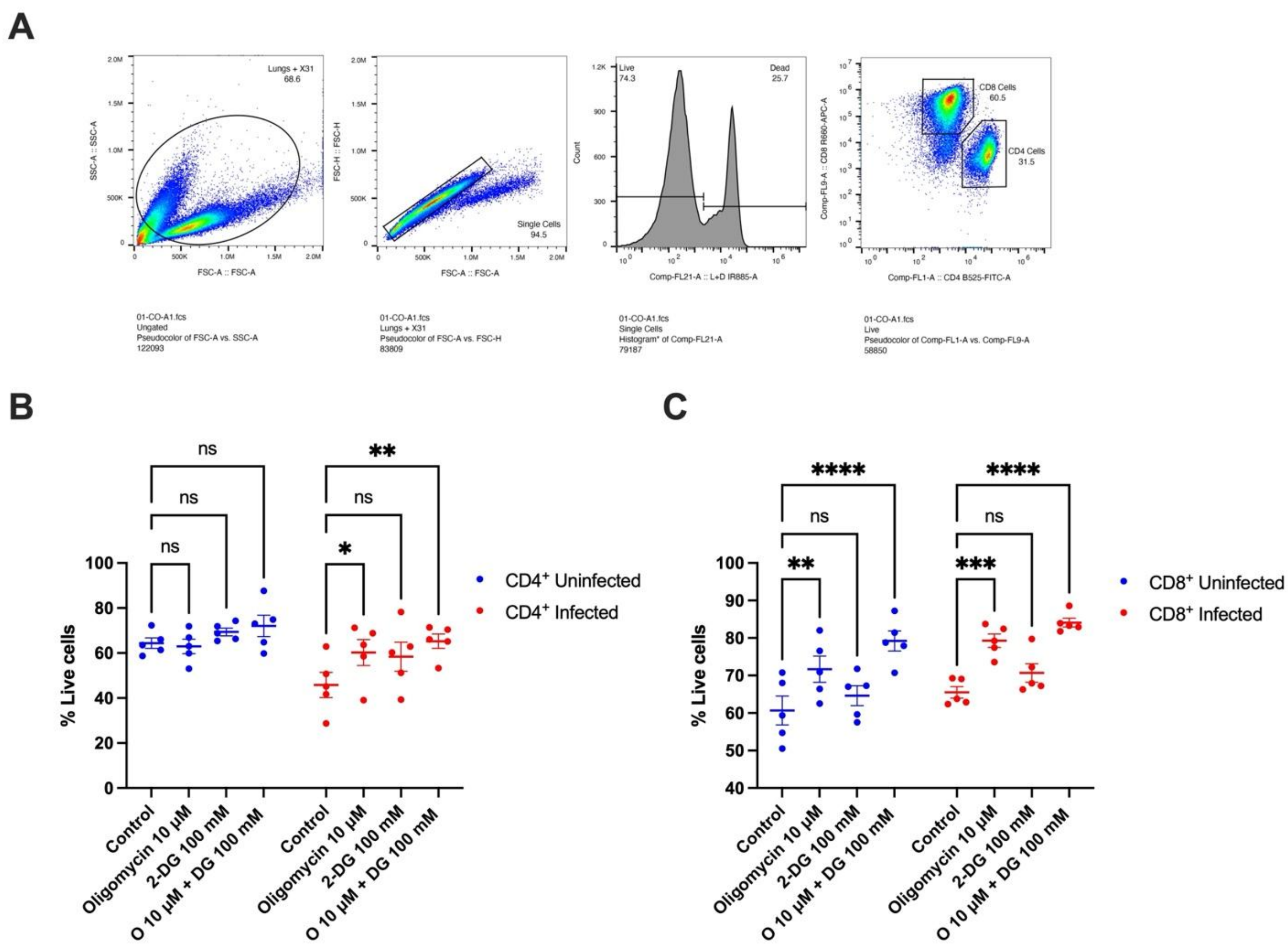

Figure S6. Gating strategy and viability controls for ATP-Red-based metabolic profiling of lung T cells from influenza-infected mice. (A) Representative flow cytometry gating strategy for ex vivo lung T cell analysis from influenza X31 infected mice. Sequential gates show selection of leukocyte-enriched events by FSC/SSC, singlet discrimination, live-cell gating using viability dye exclusion, and final identification of $CD4^+$ and $CD8^+$ T cell populations. (B) Viability of $CD4^+$ and $CD8^+$ T cells isolated from lungs of uninfected or influenza-infected mice after treatment with oligomycin, 2-DG, or oligomycin plus 2-DG under the conditions used for ATP-Red-based

metabolic dependence analysis. Baseline viability was maintained, but infected-lung T cells showed increased sensitivity to dual metabolic inhibition relative to uninfected controls. Data are shown as mean ± SD. Statistical analysis for panel B was performed using ordinary two-way ANOVA followed by multiple-comparisons testing as indicated. Panel A shows representative gating plots. ns, not significant; *$P < 0.05$; **$P < 0.01$; ***$P < 0.001$; ****$P < 0.0001$.
