# Supplementary material for: ATP-Red enables single-cell bioenergetic phenotyping by flow cytometry
Source: Cell Rep Methods. 2026 Jul 30;6(9):101537. doi: 10.1016/j.crmeth.2026.101537 (PMC13615503; doi:10.1016/j.crmeth.2026.101537)
Supplement: Supplementary file 2 [file mmc2.pdf]

Cell Reports
Methods

Article

ATP-Red enables single-cell bioenergetic phenotyping by flow cytometry

Graphical abstract

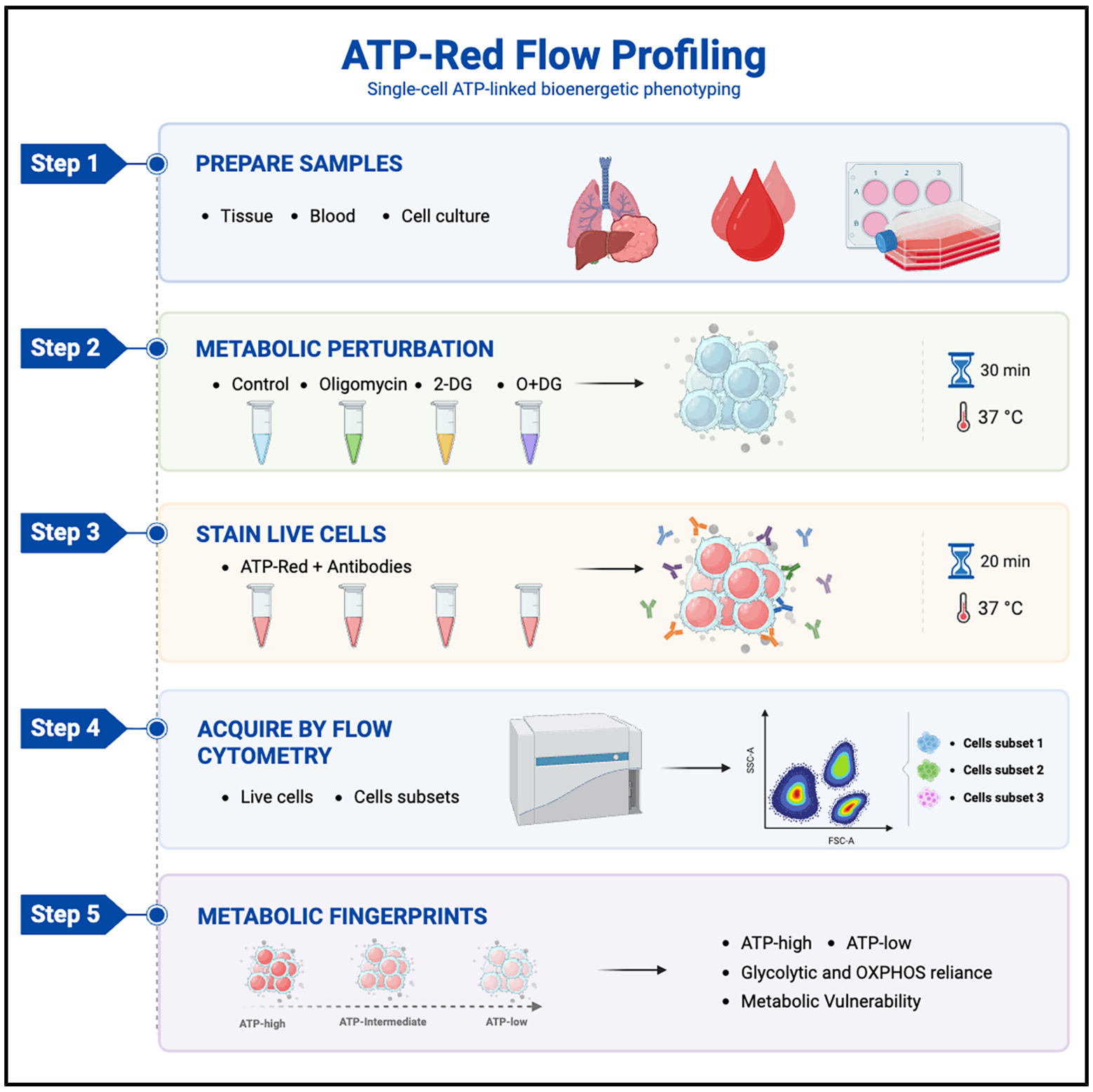

Highlights

- ATP-Red enables ATP-linked fluorescence at single-cell resolution for bioenergetics
- Benchmarking confirms ATP-Red aligns well with orthogonal ATP and flux measurements
- Detects ATP changes under ATPase inhibition, mitochondrial stress, and glycolysis
- Resolves immune cell heterogeneity when combined with immunophenotyping methods

Authors

Jose Marin, Bharati Singh, Amanda Fuchs, ..., Shannon Kruk, A. Phillip West, Peter J. McGuire

Correspondence

peter.mcguire@nih.gov

In brief

Marin et al. evaluate ATP-Red as a simple flow-cytometry-based method for measuring ATP-associated bioenergetic states in single cells. The study shows that ATP-Red captures metabolic heterogeneity, immune cell activation, and mitochondrial dysfunction, providing a practical approach for single-cell metabolic profiling in complex biological systems.

Marin et al., 2026, Cell Reports Methods *6*, 101537
September 21, 2026 Published by Elsevier Inc.
https://doi.org/10.1016/j.crmeth.2026.101537

CellPress

Cell Reports Methods

CellPress
OPEN ACCESS

Article

ATP-Red enables single-cell bioenergetic phenotyping by flow cytometry

Jose Marin,[1] Bharati Singh,[1] Amanda Fuchs,[1] Tatiana Tarasenko,[1] Emily Warren,[1,5] Martha Kirby,[2] Stacie Anderson,[2] Stephen M. Wincovitch,[3] Eliza M. Gordon-Lipkin,[1] Shannon Kruk,[1] A. Phillip West,[4] and Peter J. McGuire[1,6,*]

[1]Metabolism, Infection and Immunity Section, National Human Genome Research Institute, National Institutes of Health, Bethesda, MD, USA

[2]Flow Cytometry Core, National Human Genome Research Institute, National Institutes of Health, Bethesda, MD, USA

[3]Advanced Imaging & Analysis Core Facility, National Human Genome Research Institute, National Institutes of Health, Bethesda, MD 20892, USA

[4]The Jackson Laboratory, Bar Harbor, ME, USA

[5]Deceased

[6]Lead contact

*Correspondence: peter.mcguire@nih.gov

https://doi.org/10.1016/j.crmeth.2026.101537

MOTIVATION Current approaches for bioenergetic profiling each carry important limitations. Bulk methods such as extracellular flux analysis provide robust metabolic measurements but lack single-cell resolution and can obscure heterogeneity within complex populations. Existing flow-cytometry-based approaches, including protein translation-dependent assays, infer energetic state indirectly. Moreover, these methods may become uncoupled from intracellular ATP concentration during stress, activation, or mitochondrial dysfunction. We sought to evaluate ATP-Red as a simple, non-genetic, flow cytometry-compatible method that provides a direct ATP-associated fluorescent readout in live cells, enabling scalable single-cell bioenergetic phenotyping across diverse physiologic and pathologic contexts.

SUMMARY

Most established bioenergetic assays lack single-cell resolution and may mask metabolic heterogeneity. The main flow-cytometry-based approach for bioenergetic analysis infers energetic state from protein synthesis, although translation can become uncoupled from ATP availability under physiologic and pathologic conditions. Here, we evaluated ATP-Red as a flow-cytometry-compatible readout of energetic state at single-cell resolution. We benchmarked ATP-Red against orthogonal approaches, including colorimetric ATP quantification, the ATP/ADP biosensor PercevalHR, and extracellular flux analysis, across ATPase inhibition, glycolytic and oxidative blockade, mitochondrial dysfunction, and immune activation. Across these settings, ATP-Red tracked biologically meaningful energetic changes as a relative ATP-linked fluorescence readout. When combined with immunophenotyping, ATP-Red also captured bioenergetic heterogeneity and pathway use in T cells following activation and during influenza virus infection. These findings support ATP-Red as a practical and scalable approach for single-cell bioenergetic phenotyping.

INTRODUCTION

Cellular metabolism is a tightly regulated process that sustains essential functions such as biosynthesis, ion transport, and immune activation. At the core of this metabolic network lies adenosine triphosphate (ATP),[1] the universal energy currency that links nutrient utilization to cellular output.[2] ATP dynamics describe the spatiotemporal regulation of intracellular ATP, shaped by the balance between its production, consumption, and distribution. These dynamics reflect flux through major bioenergetic pathways, including glycolysis and oxidative phosphorylation (OXPHOS), and serve as indicators of cellular stress, dysfunction, and adaptation.[3–6]

To probe bioenergetics, several analytical techniques have been developed, each with distinct strengths and limitations in terms of resolution, throughput, and compatibility with different cell types. Genetically encoded biosensors such as PercevalHR enable dynamic, real-time monitoring of cytosolic ATP/ADP-related changes in live cells.[7,8] Despite their utility, their application in primary or fragile cells is often limited by the transfection efficiency. In addition, the fluorescent ratiometric signal can be influenced by intracellular pH, which may

This is an open access article under the CC BY license (http://creativecommons.org/licenses/by/4.0/).

complicate interpretation. Extracellular flux technologies such as the Seahorse XF analyzer are widely used to profile bulk metabolic parameters, including oxygen consumption and glycolytic activity.[9–11] However, these approaches require relatively high cell numbers and lack single-cell resolution, making them less suitable for limited or phenotypically complex samples.

Metabolic profiling at the level of single-cell resolution has advanced with SCENITH (single-cell energetic metabolism by profiling translation inhibition), a flow-cytometry-based approach that interrogates bioenergetics through the effects of metabolic perturbations on protein synthesis.[12] This strategy is particularly valuable for the analysis of complex or rare populations because it can be integrated with immunophenotyping. However, SCENITH relies on intracellular detection of puromycin incorporation after cell permeabilization, using protein synthesis as an indirect functional surrogate of cellular bioenergetics. As such, it reports a downstream consequence of the metabolic state. Growing evidence suggests that the translational activity does not always track with bioenergetics, highlighting a potential disconnect between energy state and protein synthesis under physiologic stress or mitochondrial dysfunction.[13,14] CENCAT (cellular energetics through noncanonical amino acid tagging) similarly leverages protein synthesis as a proxy for cellular metabolism while extending this framework into multi-omic contexts through compatibility with single-cell sequencing readouts.[15] Other methods include antibody-based metabolic protein profiling strategies, such as Met-Flow,[16] and genetically encoded energetic reporter platforms such as SPICE-Met.[17] This diversity highlights the need for a simple, non-genetic, flow cytometry compatible method that more directly reflects an ATP-linked energetic state in live cells.

ATP-Red is a fluorescent small-molecule sensor that binds to ATP with high specificity and provides a relative ATP-linked fluorescent readout in live cells without significant cross-reactivity to other metabolites.[18] In its inactive state, ATP-Red exists in a non-fluorescent, closed-ring conformation. Upon ATP binding, disruption of covalent interactions between boron and ribose opens the ring and generates a fluorescence signal. Although ATP-Red has been used for cellular ATP imaging, its performance characteristics, assay boundaries, and utility for systematic single-cell bioenergetic phenotyping have not been rigorously evaluated in a methodological study. Based on its chemical specificity and compatibility with standard flow cytometry, we hypothesized that ATP-Red could be used together with metabolic inhibitors and surface markers to resolve pathway use and energetic states across diverse immune and metabolic contexts.

In this study, we evaluated ATP-Red as a flow cytometry-compatible, ATP-associated readout of bioenergetics across diverse physiological and pathological contexts at single-cell resolution. Performance was benchmarked against established approaches for ATP and bioenergetic assessments, including PercevalHR, colorimetric ATP quantification, SCENITH, and extracellular flux analysis. To define its sensitivity to metabolic perturbation, ATP-Red was applied to the cells treated with inhibitors of glycolysis and OXPHOS. The probe was further used to resolve energetic phenotypes in cells with mitochondrial dysfunction and to examine metabolic remodeling during T cell activation and influenza virus infection. Together, we tested whether ATP-Red could provide a practical and scalable approach for bioenergetic phenotyping when interpreted alongside established orthogonal metabolic assays.

RESULTS

ATP-Red reports whole-cell ATP state and responds to metabolic perturbations

ATP is primarily generated by OXPHOS in mitochondria and by glycolysis in the cytoplasm, with intracellular distribution varying with the metabolic state.[19,20] To determine how ATP-Red should be interpreted at the cellular level, we first examined its intracellular localization in Madin-Darby canine kidney (MDCK) cells by spinning-disk confocal microscopy. The cells were stained with Hoechst 33342, MitoTracker Green FM, CellTracker, and ATP-Red. ATP-Red fluorescence was broadly distributed throughout the cell, with signal present in both mitochondrial and non-mitochondrial regions (Figure 1A). Quantitative co-localization analysis did not reveal significant enrichment of ATP-Red within the cytosolic or mitochondrial compartments, supporting its interpretation as a whole-cell ATP-associated fluorescence readout, rather than a compartment-restricted reporter.

To benchmark ATP-Red against established indicators of the cellular energy status, we compared its response to that of three complementary sensors of ATP or functional energy availability (Figure 1B). As a model system, we induced ATP accumulation in the MDCK cells by inhibiting two major ATP-consuming enzymes, Na^+/K^+-ATPase and Ca^{2+}-ATPase, using ouabain and thapsigargin, respectively. Inhibiting these pumps is expected to reduce ATP hydrolysis and increase intracellular ATP levels. As the first comparator, we measured cytosolic energy charge using PercevalHR, a genetically encoded fluorescent biosensor that reports the ATP:ADP ratio.[7,8] In untreated cells, the ATP:ADP ratio was approximately 1.0, whereas inhibitor treatment elevated this value to over 1.5, consistent with the reduced ATP turnover (Figure 1C). We next quantified total ATP, using a standard colorimetric assay, which showed a measurable increase in response to both inhibitors, with levels rising by ∼25%–40% compared with controls (Figure 1D). We then applied ATP-Red to assess ATP-associated fluorescence under these conditions by flow cytometry. Fluorescence intensity increased in the treated cells by ∼10%–70% (Figure 1E), indicating that ATP-Red can detect ATP accumulation in response to decreased consumption. Cell viability remained above 94% across all treatment groups (Figures S1A and S1B), ruling out cytotoxicity as a confounding factor.

Having established that ATP-Red reports whole-cell ATP state under reduced consumption, we next asked how this signal responds to compartment-specific constraints on ATP exchange. Mitochondrial adenine nucleotide transport was disrupted using bongkrekic acid (BKA), an inhibitor of the adenine nucleotide translocase (ANT). To establish the metabolic effects of ANT inhibition as a reference for ATP-Red measurements, MDCK cells were first pre-incubated with BKA (2.5, 5, or 10 μM) for 60 min and subjected to the extracellular flux analysis. Under these conditions, BKA markedly reduced basal respiration (Figure 1F) and ATP-linked respiration (Figure 1G), with a concomitant increase in glycolysis at higher concentrations (Figure 1H). These findings were consistent with a bioenergetic shift away from OXPHOS

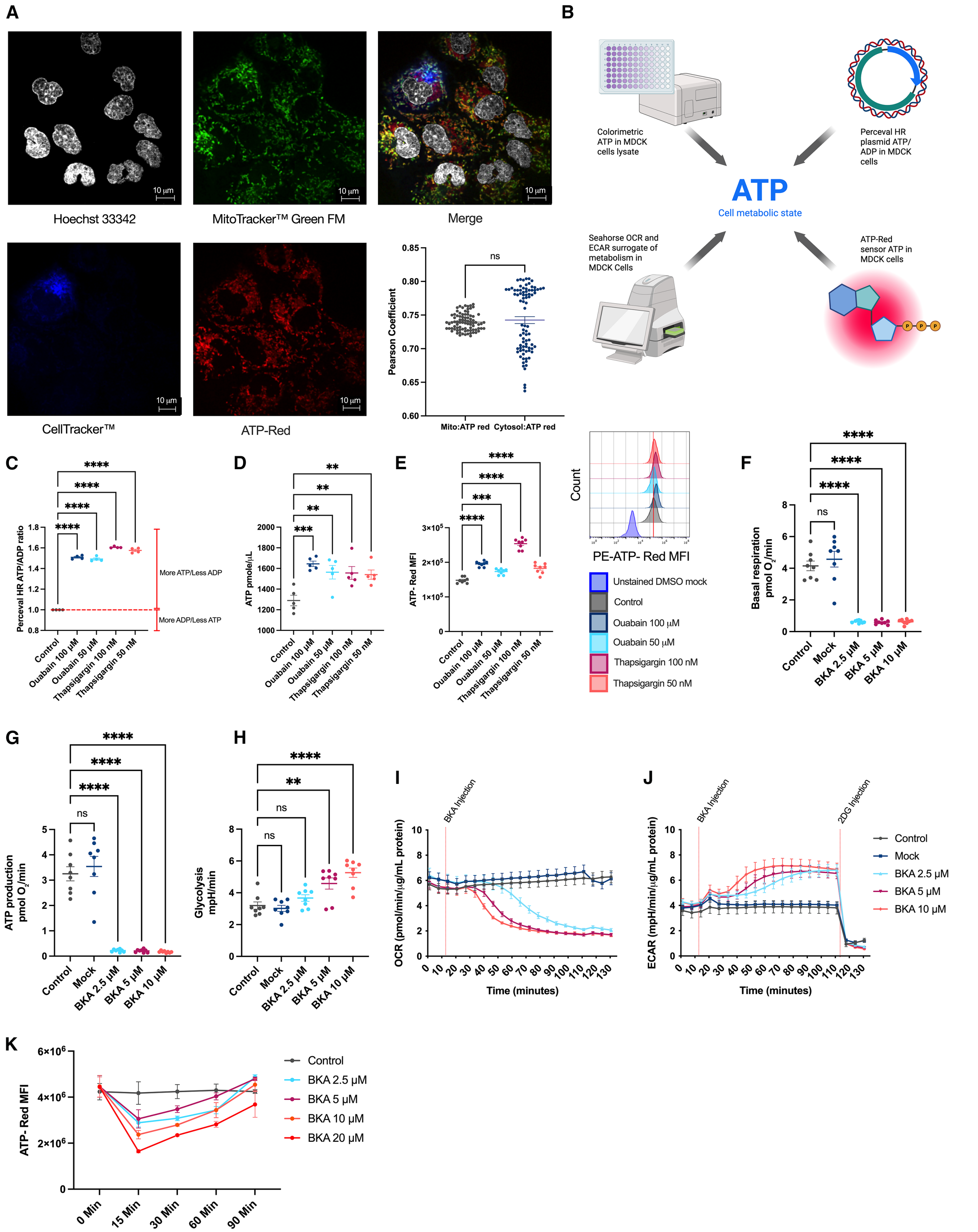

(legend on next page)

toward glycolytic compensation. Representative oxygen consumption rate (OCR) and extracellular acidification rate (ECAR) traces are shown in Figures S1C and S1D, respectively. To define the kinetics of this response, we performed separate acute experiments in which BKA (2.5, 5, or 10 μM) was injected during acquisition, and OCR and ECAR were monitored dynamically in real time. In these acute analyses, BKA elicited a rapid, concentration-dependent decrease in OCR, consistent with a disruption of OXPHOS beginning at about 30 min (Figure 1I). Similar to our pre-treatment studies, ECAR increased in a reciprocal manner, indicating a rapid compensatory shift toward glycolytic metabolism (Figure 1J). Importantly, this BKA-induced increase in ECAR was attenuated after the addition of 2-deoxy-D-glucose (2-DG), demonstrating that the acidification response was glycolytic in origin.

Following extracellular flux characterization of BKA treatment, we assessed the temporal response of ATP-Red. BKA (2.5, 5, or 10 μM) induced a decrease in ATP-Red fluorescence, most evident at the 15 min time point, with approximate reductions of 35%–47%, respectively, relative to baseline (Figure 1K). This initial decline was followed by a marked recovery or upward trend at later time points. By 90 min, ATP-Red levels had largely recovered. This biphasic response is consistent with the compensatory adaptation seen in our extracellular flux analysis. Importantly, viability remained broadly stable during these short-term treatments (Figure S1E), indicating that Figure 1K reflects metabolic remodeling rather than acute cell loss.

ATP-Red detects ATP depletion during glycolytic inhibition

Building on our validation across models of ATP perturbation, we next assessed ATP-Red performance under targeted glycolytic inhibition. Glycolysis was inhibited in MDCK cells using 2-DG, a competitive glucose analog that is phosphorylated to 2-DG-6-phosphate, thereby stalling the glycolytic flux, blocking substrate-level phosphorylation, and reducing ATP production from glucose oxidation (Figure 2A). The cells were exposed to increasing concentrations of 2-DG, and relative ATP levels were determined using ATP-Red alongside complementary methods.

PercevalHR detected concentration-dependent declines in the ATP:ADP ratio to 53.0%, 40.0%, and 24.3% of control levels at 50, 100, and 200 mM 2-DG, respectively (Figure 2B). As an orthogonal measurement of total ATP, a colorimetric assay performed on cell lysates showed a similar dose-dependent reduction across all treatment conditions (Figure 2C). With ATP-Red, the fluorescence intensity progressively declined to 65.8%, 49.0%, and 43.4% of control at 50, 100, and 200 mM, respectively (Figure 2D). Cell viability remained above 96% across all conditions (Figures S2A and S2B).

ATP-Red detects ATP depletion following mitochondrial ATP synthase inhibition

Because mitochondrial OXPHOS is the primary source of cellular ATP, we next tested ATP-Red under conditions of selective inhibition of this pathway. MDCK cells were treated with oligomycin, a specific ATP synthase (complex V) inhibitor that blocks proton translocation and prevents ATP synthesis at the terminal step of the electron transport chain (Figure 3A). ATP levels were then quantified using ATP-Red alongside two reference assays.

PercevalHR reported a dose-dependent reduction in the cytosolic ATP:ADP ratio (Figure 3B). Consistently, colorimetric measurement of total ATP showed declines to 91.9%, 82.3%, and 70.6% of control levels at 5, 10, and 20 μM, respectively

Figure 1. Comparison of ATP-associated and ATP-related readouts of cellular metabolic state in MDCK cells

(A) Spinning-disk confocal microscopy of MDCK cells stained with Hoechst 33342, MitoTracker Green FM, CellTracker, and ATP-Red. Merged images show broad intracellular distribution of ATP-Red with signal overlapping both mitochondrial and non-mitochondrial intracellular regions. Pearson's correlation analysis showed no significant difference between ATP-Red co-localization with mitochondrial and cellular markers, supporting the interpretation of ATP-Red as a whole-cell ATP-associated fluorescence readout, rather than a compartment-restricted signal. Scale bars, 10 μm.

(B) Schematic representation of four complementary approaches used to assess ATP-related energetic state in MDCK cells: colorimetric ATP quantification from cell lysates, PercevalHR biosensor measurement of ATP/ADP-related changes, OCR and ECAR by extracellular flux analysis, and ATP-Red fluorescence measurement by flow cytometry.

(C) PercevalHR ATP/ADP ratio readout in MDCK cells treated with inhibitors of ATP consumption, ouabain and thapsigargin, at the indicated concentrations. All treatment conditions showed increased ATP/ADP ratio relative to control.

(D) Total intracellular ATP quantified by colorimetric assay in MDCK cells treated with ouabain or thapsigargin. Inhibitor-treated conditions showed increased ATP relative to control.

(E) ATP-Red fluorescence measured by flow cytometry in MDCK cells treated with ouabain or thapsigargin. All inhibitor-treated conditions showed increased ATP-Red fluorescence relative to the control. Representative histogram overlays are shown at the right.

(F) Basal respiration measured by extracellular flux analysis in MDCK cells pretreated with bongkrekic acid (BKA) at the indicated concentrations.

(G) ATP-linked respiration in BKA-treated MDCK cells derived from Mito Stress analysis.

(H) Glycolysis in BKA-treated MDCK cells derived from glycolysis stress analysis.

(I) Representative OCR traces from acute extracellular flux analysis of MDCK cells after injection of BKA at the indicated concentrations. BKA induced a rapid, dose-dependent decrease in oxygen consumption, consistent with impaired mitochondrial ATP-linked respiration.

(J) Representative ECAR traces from acute extracellular flux analysis of MDCK cells after injection of BKA at the indicated concentrations. BKA induced a reciprocal increase in extracellular acidification, consistent with compensatory glycolytic engagement.

(K) ATP-Red MFI (mean fluoresence intensity) in MDCK cells exposed to increasing concentrations of BKA for the indicated times.

Data are shown as mean ± SEM, unless otherwise indicated; data in (K) are shown as mean ± SD. Individual data points represent independent biological replicates or independent measurements, as appropriate for the experimental design. Technical replicates, when present, were averaged before statistical analysis. Statistical analysis for (C–H) was performed using ordinary one-way ANOVA followed by multiple-comparisons testing against control, unless otherwise indicated, and for (K), ordinary two-way ANOVA was used, with treatment and exposure time as factors, followed by Dunnett's multiple-comparisons test comparing each BKA condition with control at each time point. ns, not significant; *$p < 0.05$; **$p < 0.01$; ***$p < 0.001$; ****$p < 0.0001$.
See also Figure S1.

CellPress
OPEN ACCESS

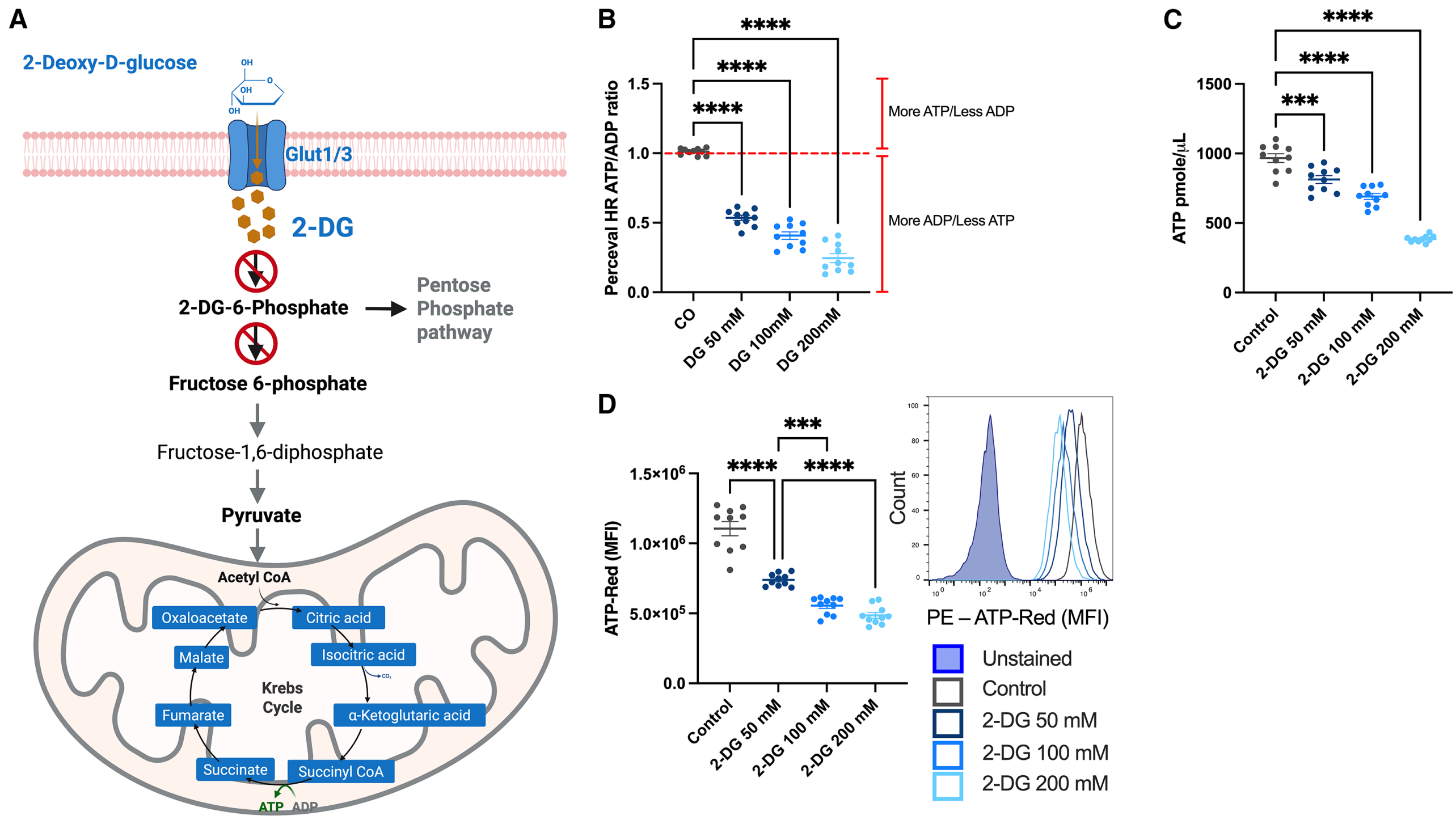

Figure 2. Inhibition of glycolysis by 2-DG reduces ATP availability in MDCK cells

(A) Schematic of 2-DG uptake and metabolic inhibition. 2-DG is transported into the cell via GLUT1/3 and phosphorylated to 2-DG-6-phosphate, which inhibits glycolysis by blocking conversion to fructose-6-phosphate and downstream metabolites, limiting entry into the pentose phosphate and glycolytic pathways. This leads to reduced pyruvate and Krebs cycle input, ultimately impairing ATP production.

(B) PercevalHR-based measurement of ATP/ADP ratio in MDCK cells treated with increasing concentrations of 2-DG. All concentrations tested significantly reduced the ATP/ADP ratio compared with control.

(C) Quantification of total intracellular ATP using a colorimetric assay. ATP levels decreased significantly with 2-DG treatment.

(D) Flow cytometry analysis of ATP-associated fluorescence using ATP-Red. Fluorescence intensity decreased significantly with 2-DG treatment.

Data are shown as mean ± SEM. Individual data points represent independent biological replicates or independent measurements, as appropriate for the experimental design. Technical replicates, when present, were averaged before statistical analysis. Statistical analysis was performed using ordinary one-way ANOVA followed by multiple-comparisons testing against control. ns, not significant; *$p < 0.05$; **$p < 0.01$; ***$p < 0.001$; ****$p < 0.0001$.

See also Figure S2.

(Figure 3C). Analysis with ATP-Red revealed parallel decreases in fluorescence, reaching 86.7%, 77.0%, and 69.0% of control across the same concentrations (Figure 3D). Cell viability remained above 97% in all treatment groups (Figures S3A and S3B).

ATP-Red reveals energetic deficits and metabolic reprogramming in NDUFS4-deficient cells

To extend our evaluation of ATP-Red beyond defined chemical perturbations, we applied it to a genetic model of mitochondrial disease. NDUFS4 is a key subunit of mitochondrial complex I, with its deficiency producing OXPHOS impairment. NDUFS4-knockout MDCK cells were generated using CRISPR-Cas9-mediated gene editing. To confirm that NDUFS4-KO cells exhibited mitochondrial dysfunction and bioenergetic rewiring, we performed extracellular flux analysis. KO cells showed reduced basal respiration and ATP-linked respiration relative to wild-type (WT) controls, consistent with compromised OXPHOS (Figures 4A, 4B, and 4D). In parallel, KO cells displayed increased extracellular acidification, supporting a shift toward glycolytic metabolism (Figures 4C and 4E). No glycolytic reserve capacity was observed, consistent with the cells already operating near maximal glycolytic activity.

To extend these findings beyond flux-based measurements, we next examined orthogonal ATP-related readouts. NDUFS4-KO cells exhibited a reduced ATP/ADP ratio measured with PercevalHR (Figure 4F) and a lower total ATP content by colorimetric assay (Figure 4G), confirming impaired energetic balance. ATP-Red flow cytometry independently confirmed lower ATP-associated fluorescence in KO cells at the single-cell level (Figure 4H).

Because SCENITH-based approaches rely on active protein synthesis as a functional readout, we were concerned that impaired translation in NDUFS4-KO cells could confound interpretation. We, therefore, assessed the translational capacity directly. Protein synthesis, measured by Click-iT HPG (L-homopropargylglycine) incorporation, was significantly reduced in KO cells relative to WT controls (Figure 4I). The sensitivity of this assay to biosynthetic inhibition was

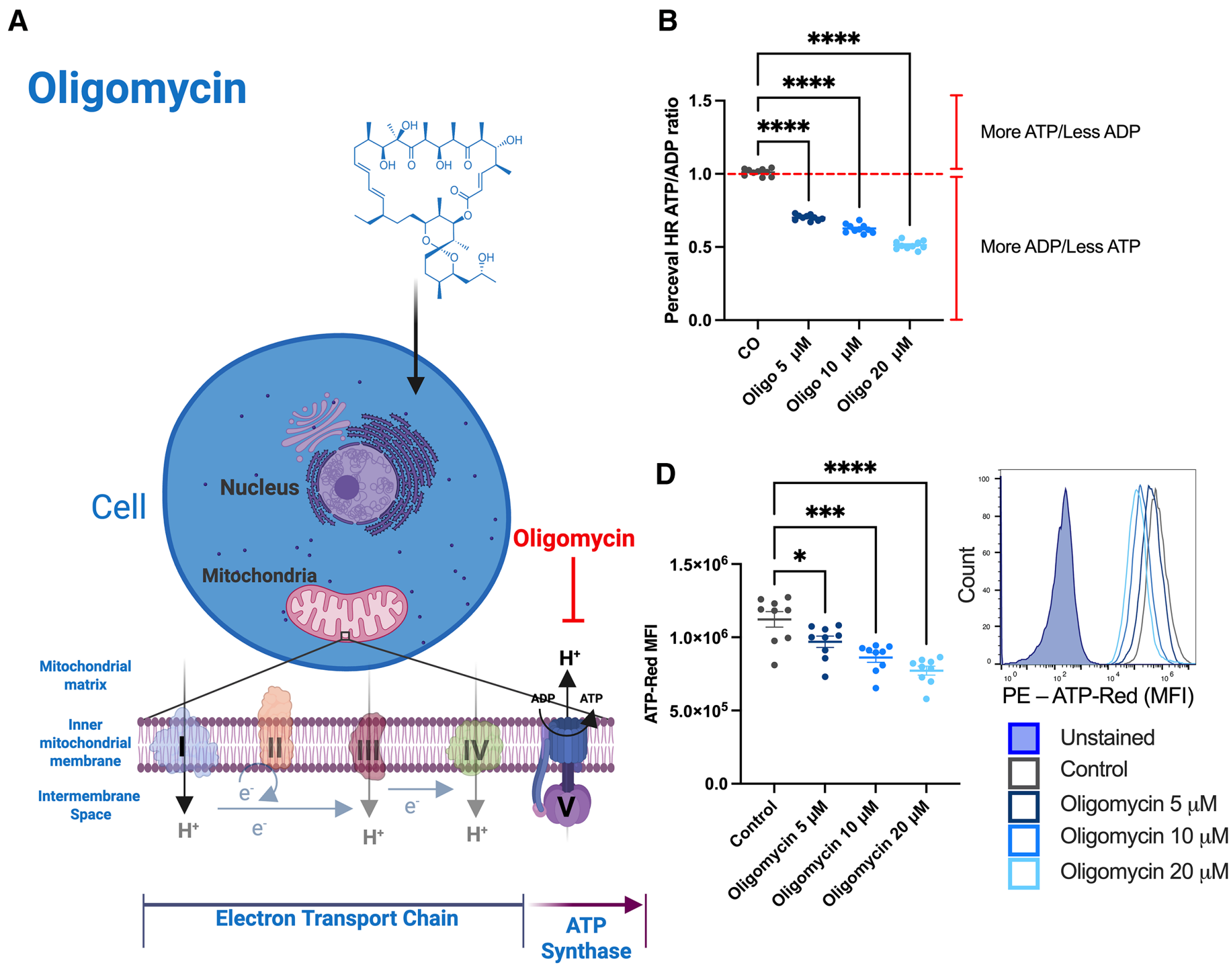

Figure 3. Inhibition of mitochondrial ATP production by oligomycin impairs the cellular energy state in MDCK cells

(A) Diagram illustrating the mechanism of oligomycin, an inhibitor of ATP synthase, complex V, in the mitochondrial electron transport chain. By blocking proton flow through ATP synthase, oligomycin prevents mitochondrial ATP production despite an intact electron transport chain and proton gradient.

(B) ATP/ADP ratio measured with the PercevalHR biosensor. Oligomycin treatment led to a dose-dependent decrease in ATP/ADP ratio compared with control.

(C) Total intracellular ATP quantified using a colorimetric assay. ATP levels decreased following oligomycin treatment, with significant reductions at the indicated concentrations.

(D) ATP-associated fluorescence measured by ATP-Red flow cytometry. ATP-Red signal decreased significantly with oligomycin treatment, consistent with the impaired mitochondrial ATP production.

Data are shown as mean ± SEM. Individual data points represent independent biological replicates or independent measurements, as appropriate for the experimental design. Technical replicates, when present, were averaged before statistical analysis. Statistical analysis was performed using ordinary one-way ANOVA followed by multiple-comparisons testing against control. ns, not significant; *$p < 0.05$; **$p < 0.01$; ***$p < 0.001$; ****$p < 0.0001$.

See also Figure S3.

confirmed using puromycin and cycloheximide, which produced similar reductions (Figure S4B). Importantly, basal viability did not differ between the WT and NDUFS4-KO cells (Figure S4A), indicating that the reduced HPG signal in KO cells was not attributable to overt loss of viability.

To determine how this baseline defect impacts SCENITH-based metabolic profiling, we next applied inhibitor-based analysis within an SCENITH framework[12] and compared translation- and ATP-based readouts under identical perturbations. In WT cells, the anti-puromycin signal decreased as expected following oligomycin, 2-DG, and combined treatment, whereas NDUFS4-KO cells began from a reduced baseline and exhibited a compressed dynamic range across conditions (Figure 4J). By contrast, ATP-Red yielded a broader and more physiologically coherent metabolic profile (Figure 4K). ATP-Red-based analysis revealed markedly reduced OXPHOS dependence together with increased glycolytic dependence and preserved glycolytic capacity in the KO cells, consistent with a shift away from mitochondrial ATP production toward compensatory glycolytic support. These ATP-derived profiles closely matched the extracellular flux phenotype of reduced OCR and elevated ECAR in KO cells (Figures 4A–4E), reinforcing the conclusion that NDUFS4 deficiency drives metabolic rewiring in MDCK cells. Consistent with this interpretation, viability remained broadly preserved under oligomycin, 2-DG, and combined treatment conditions, with only modest changes (Figures S4C and S4D). Our findings highlight a key limitation of translation-based metabolic readouts in models with OXPHOS dysfunction: when protein synthesis is reduced at baseline, puromycin-based measurements exhibit a compressed dynamic range and reduced sensitivity to bioenergetic perturbations. Because SCENITH-derived metrics rely on relative changes in translation, this compression can distort inferred pathway contributions, including overestimation of OXPHOS dependence despite impaired mitochondrial ATP production.

CellPress
OPEN ACCESS

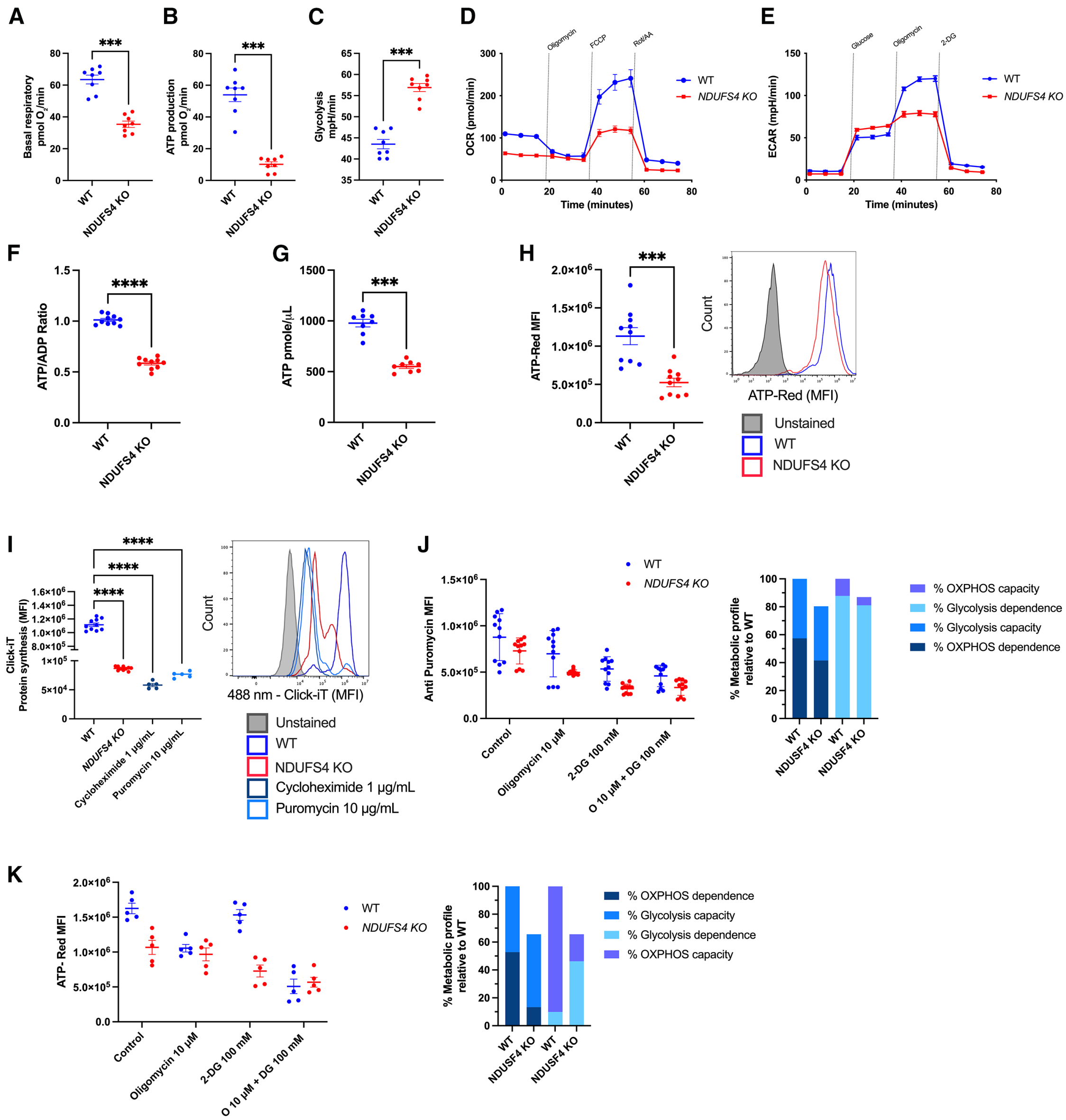

Figure 4. ATP-Red identifies energetic deficits and metabolic rewiring in NDUFS4-deficient cells

(A) Basal oxygen consumption rate (OCR) in WT and NDUFS4-KO MDCK cells, measured by extracellular flux analysis. KO cells showed reduced basal respiration relative to WT cells.

(B) ATP-linked respiration in WT and NDUFS4-KO MDCK cells derived from Mito Stress analysis. KO cells showed reduced ATP-linked respiration compared with WT cells.

(C) Glycolytic activity in WT and NDUFS4-KO MDCK cells derived from extracellular flux analysis. KO cells showed increased glycolytic activity relative to WT cells.

(D) Representative OCR traces from WT and NDUFS4-KO MDCK cells during Mito Stress analysis following sequential injection of oligomycin, carbonyl cyanide p-trifluoromethoxyphenylhydrazone (FCCP), and rotenone/antimycin A.

(E) Representative ECAR traces from WT and NDUFS4-KO MDCK cells showing increased extracellular acidification in KO cells, consistent with greater glycolytic engagement.

(F) Cytosolic ATP/ADP ratio measured with PercevalHR in WT and NDUFS4-KO cells. KO cells showed a reduced ATP/ADP ratio relative to WT cells.

(G) Total ATP content measured by colorimetric assay in WT and NDUFS4-KO cells. KO cells showed reduced ATP content compared with WT cells.

(H) ATP-associated fluorescence measured by ATP-Red in WT and NDUFS4-KO cells. KO cells showed reduced ATP-Red fluorescence relative to WT cells. Representative histogram overlays are shown at right.

(I) Protein synthesis measured by Click-iT HPG incorporation in WT and NDUFS4-KO cells. KO cells showed reduced HPG incorporation relative to WT cells. Representative histogram overlays are shown at right.

(J) Anti-puromycin fluorescence intensity in WT and NDUFS4-KO cells treated with oligomycin (O), 2-deoxy-D-glucose (2-DG), or combined oligomycin plus 2-DG (ODG). KO cells started from a lower basal anti-puromycin signal and remained lower across perturbation conditions. Right: SCENITH metabolic profiling derived

(legend continued on next page)

ATP-Red resolves subset-specific metabolic remodeling during T cell activation

Given the strong performance of ATP-Red across perturbation models, we next applied it as the primary metabolic readout to exploit its single-cell resolution. As a physiological test case, we examined T cell activation, a process driven by extensive bioenergetic reprogramming to meet the demands of clonal expansion and effector function. Because ATP-Red is fully compatible with flow cytometry, it enables ATP-associated fluorescence measurement in parallel with surface marker staining, allowing metabolic profiling of defined immune subsets rather than bulk populations. This feature makes ATP-Red particularly useful for immunometabolic analysis in heterogeneous samples.

T cells were isolated from the spleens of C57BL/6 mice and stimulated with anti-CD3/CD28. CD4$^+$ and CD8$^+$ T cells showed a 25%–30% increase in ATP-associated fluorescence (Figures 5A and 5C), coinciding with robust induction of the activation markers CD25, CD44, and CD69 (Figures 5B–5D). To determine how activation reshaped pathway use, resting and activated T cells were treated with oligomycin, 2-DG, or both, followed by ATP-Red measurement. Metabolic dependence analysis revealed that in CD4$^+$ T cells, activation increased glycolytic dependence by 38.9% and glycolytic capacity by 61.1%, while decreasing OXPHOS dependence modestly by 11.0%. CD8$^+$ T cells exhibited a stronger shift, with glycolytic dependence rising by 70.1%, glycolytic capacity by 133.4%, and OXPHOS dependence falling by 33.4%. Despite this reduced relative reliance on mitochondrial ATP production, OXPHOS capacity remained high in both subsets (Figures 5E and 5F, right), consistent with preserved metabolic flexibility.

To test whether selective survival contributed to these patterns, we examined viability across all conditions. Activated cells, particularly those expressing CD25 and CD69, maintained equal or greater viability than their resting counterparts (Figures S5A–S5D). Even under dual inhibition, CD8$^+$ T cells showed superior survival. Overall, ATP-Red detects activation-associated increases in ATP-linked signal and resolves subset-specific pathway engagement and metabolic flexibility within activated CD4$^+$ and CD8$^+$ T cell populations.

ATP-Red reveals metabolic rewiring in lung T cells during infection

To determine whether ATP-Red can profile metabolic changes *ex vivo* under pathologic conditions, we applied it to T cells isolated directly from the lungs of mice infected with influenza A virus (IAV). This model captures immune activation within a physiologic tissue environment, where local inflammation and stress can shape bioenergetic remodeling. In this setting, ATP-Red allowed metabolic interrogation of defined T cell subsets *ex vivo* without the need for transfection, reporter expression, or bulk population averaging.

T cells were isolated from lung suspensions five days after infection with the X31 strain of IAV and compared with uninfected controls (Figure 6A). T cell subsets were defined using the gating strategy shown in Figure S6A. Infected lungs showed marked T cell recruitment, with CD4$^+$ T cells increasing from 7.0% to 32.9% and CD8$^+$ T cells from 13.3% to 23.9% of total live cells (Figure 6B). Viability remained stable with infection (Figure 6C). ATP-Red fluorescence increased in both subsets, consistent with activation-associated metabolic demand, with CD4$^+$ T cells showing a 21.3% increase in ATP-associated fluorescence and CD8$^+$ T cells showing a 69.3% increase (Figure 6D).

To evaluate pathway engagement, we treated *ex vivo* lung T cells with oligomycin, 2-DG, or both, followed by ATP-Red quantification and metabolic dependence analysis as above. As observed *in vitro* (Figure 5), infection shifted pathway reliance toward glycolysis. In CD4$^+$ T cells, OXPHOS dependence decreased by 22.2%, whereas glycolytic dependence increased by 1,043.5%. Glycolytic capacity rose by 91.1%, and OXPHOS capacity dropped by 96.2%. CD8$^+$ T cells showed a more modest relative reprogramming, with a 1.1% decrease in OXPHOS dependence, a 93.4% increase in glycolytic dependence, a 44.8% increase in glycolytic capacity, and a 38.4% reduction in OXPHOS capacity (Figures 6E and 6F). These results are broadly consistent with the metabolic remodeling observed during *in vitro* T cell activation, namely increased glycolytic engagement together with altered balance between the two major ATP-generating pathways.

Although baseline viability was maintained, T cells from infected lungs were significantly more sensitive to dual metabolic inhibition than those from uninfected controls (Figures S6B and S6C), indicating that activation *in vivo* imposes greater energetic strain. These observations were consistent across replicates and supported by standard flow cytometry gating strategies (Figure S6A).

DISCUSSION

In this study, we position ATP-Red as a practical complementary addition to the current bioenergetic toolbox. Bulk metabolic

from puromycin incorporation after treatment with oligomycin, 2-DG, or ODG. In WT cells, inhibitor responses resolved the expected relative contributions of mitochondrial and glycolytic function to the maintenance of protein synthesis. In NDUFS4-KO cells, the puromycin-based profile was compressed by reduced basal protein synthesis and is, therefore, interpreted as reflecting the ability to sustain biosynthetic output under energetic stress rather than directly resolving pathway-specific energetic contribution.

(K) ATP-Red-based metabolic profiling under the same inhibitor conditions. ATP-Red-derived estimates indicated reduced OXPHOS-associated ATP contribution together with increased glycolytic reliance in NDUFS4 KO cells, consistent with the extracellular flux measurements shown in (A–E).

Data are shown as mean ± SEM. Individual data points represent independent biological replicates or independent measurements, as appropriate for the experimental design. Technical replicates, when present, were averaged before statistical analysis. Statistical analysis for (A–C) and (F–I) was performed using unpaired two-tailed tests or ordinary one-way ANOVA followed by multiple-comparisons testing, as appropriate. For (J and K), statistical analysis was performed using ordinary two-way ANOVA with genotype and treatment as factors, followed by multiple-comparisons testing. ns, not significant; *$p < 0.05$; **$p < 0.01$; ***$p < 0.001$; ****$p < 0.0001$.

See also Figure S4.

CellPress
OPEN ACCESS

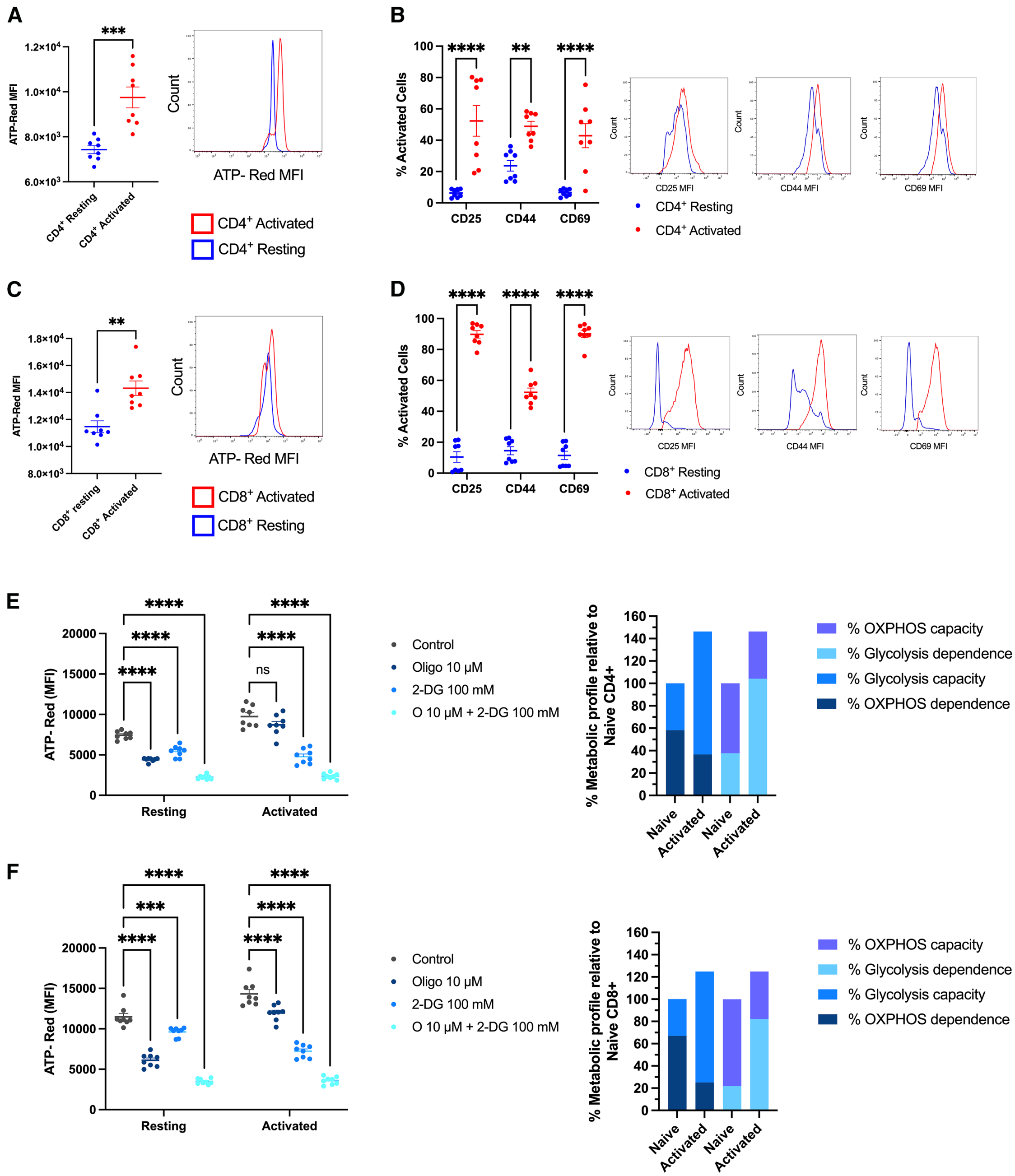

Figure 5. Activation of CD4$^+$ and CD8$^+$ T cells increases ATP-associated fluorescence and alters metabolic dependence

(A) ATP-associated fluorescence measured by ATP-Red in CD4$^+$ T cells. Activated CD4$^+$ T cells showed significantly increased ATP-Red fluorescence compared with non-activated cells. Right: representative histogram overlay of ATP-Red signal.

(B) Flow cytometry analysis of the activation markers CD25, CD44, and CD69 confirms robust activation in CD4$^+$ T cells. Right: representative histograms showing increased expression of activation markers in activated versus non-activated CD4$^+$ cells.

(C) ATP-associated fluorescence measured by ATP-Red in CD8$^+$ T cells. Activation resulted in a significant increase in ATP-Red fluorescence. Right: representative histogram overlay showing rightward shift in fluorescence.

(D) Activation marker expression in CD8$^+$ T cells increased upon activation. Right: representative histograms of activation marker expression.

(E) Metabolic profiling of CD4$^+$ T cells using ATP-Red under basal and inhibitor-treated conditions. Activated cells showed increased ATP-associated fluorescence under control and oligomycin-treated conditions, whereas combined oligomycin plus 2-DG treatment markedly reduced ATP-Red signal in both non-activated and activated cells. Right: inferred metabolic profile showing relative pathway dependence and capacity.

(F) Metabolic profiling of CD8$^+$ T cells using ATP-Red under basal and inhibitor-treated conditions. Activated cells showed increased ATP-associated fluorescence under control and oligomycin-treated conditions, whereas combined oligomycin plus 2-DG treatment markedly reduced ATP-Red signal. Right: inferred metabolic profile consistent with altered pathway reliance following activation.

(legend continued on next page)

assays such as extracellular flux analysis provide powerful measurements of pathway activity but lack single-cell resolution, whereas translation-based flow cytometry methods infer energetic state through downstream functional consequences, rather than ATP-linked signal itself. ATP-Red addresses this gap by providing a non-genetic, flow cytometry-compatible, and relative ATP-associated readout that can be deployed in heterogeneous populations without transfection or stable reporter expression. Across pharmacologic, genetic, and immunologic models, ATP-Red tracked biologically meaningful energetic remodeling when interpreted alongside orthogonal assays, including PercevalHR, colorimetric ATP quantification, and extracellular flux analysis.

Protein synthesis does not uniformly track with the cellular energetic state, particularly under conditions of mitochondrial dysfunction or stress. This principle was evident in NDUFS4-deficient MDCK cells where ATP-Red identified reduced OXPHOS, elevated glycolytic compensation, a depressed ATP/ADP ratio, and impaired biosynthesis. Although these cells retained basal viability, they failed to sustain ATP-linked signal or protein synthesis under stress, consistent with known complex I deficiency phenotypes.[21,22] In NDUFS4-deficient cells, SCENITH-derived estimates of OXPHOS dependence were inflated by the low basal translation state and compressed dynamic range of the puromycin readout, leading to an apparent overestimation of mitochondrial pathway reliance relative to ATP-Red and extracellular flux measurements. This mismatch highlights a broader limitation of translation-based metabolic readouts, potentially yielding misleading interpretations (24–26). Our findings, therefore, support the view that protein synthesis-based assays can underestimate metabolic potential in diseased or stressed states,[1,23,24] whereas ATP-Red provides a more direct and consistent readout of ATP-associated energetic dynamics, revealing both resilience and vulnerability.

Dissociation between ATP abundance and biosynthetic output supports a model in which cells enter a protective, energy-conserving state under conditions of restricted consumption.[25] Such behavior is consistent with the concept of metabolic allostasis, in which energetic balance is actively regulated, rather than being passively maintained.[5,26] Similar adaptations are observed in the integrated stress response, which suppresses global protein synthesis to conserve energy and restore homeostasis,[27] and in ischemic preconditioning, where limiting energy expenditure renders myocardium more resistant to subsequent injury.[28] Together, these precedents underscore that down-regulation of biosynthesis in the presence of preserved ATP represents a conserved protective strategy, rather than simple metabolic failure.

A major strength of ATP-Red in this study is that it could be interpreted across multiple orthogonal frameworks. PercevalHR provided a genetically encoded validation of ATP/ADP-related changes, colorimetric assays supported biochemical trends in ATP abundance, and extracellular flux analysis independently resolved changes in mitochondrial respiration and glycolytic compensation. Taken together, these complementary approaches indicate that ATP-Red should not be viewed as a replacement for flux analysis or engineered biosensors, but rather as a practical single-cell assay that gains power when integrated with the established metabolic methods. In this context, ATP-Red offers a useful balance of accessibility, throughput, and biological resolution.

Together, our findings establish ATP-Red as a robust, flow cytometry-compatible probe for measuring relative ATP-associated fluorescence at single-cell resolution. By providing a live-cell ATP-associated readout at single-cell resolution, ATP-Red overcomes key limitations of surrogate assays and reveals metabolic flexibility and vulnerability across the genetic, pharmacologic, and physiologic contexts. The consistency of ATP-Red readouts with functional metabolic outputs highlights its utility as part of multi-parameter profiling strategies. Importantly, the ability to integrate ATP measurements into high-dimensional flow cytometry opens the door to applications such as energy-based cell sorting, metabolic phenotyping, and multi-omic analysis, offering precision in the study of immunometabolism and beyond.

Limitations of the study

Across conditions, ATP-Red changes in viable cells were moderate in magnitude: $\sim$10%–50%, with larger transient shifts in selected perturbations. These changes occurred over short timescales and in the setting of preserved viability, consistent with metabolic adaptation, rather than catastrophic ATP depletion. ATP-Red also provides a relative fluorescence-based readout, rather than an absolute measure of intracellular ATP. Its signal should, therefore, be interpreted alongside orthogonal assays and with consideration of factors such as probe loading, compartmental distribution, and metabolic state. Consistent with our imaging data, ATP-Red is best interpreted as a whole-cell ATP-associated readout, rather than a compartment-specific reporter. Further benchmarking across additional cell types and primary samples is important to define the generalizability and limits of this approach.

RESOURCE AVAILABILITY

Lead contact

Requests for further information, resources, and reagents should be directed to and will be fulfilled by the lead contact, Peter J. McGuire (peter.mcguire@nih.gov).

Materials availability

All unique/stable reagents generated in this study are available from the lead contact, with a completed material(s) transfer agreement.

Data are shown as mean ± SEM. Individual data points represent independent mouse-derived splenocyte preparations or independent measurements, as appropriate for the experimental design. Technical replicates, when present, were averaged before statistical analysis. ATP-Red and activation-marker fluorescence values were quantified by flow cytometry from gated live singlet CD4$^+$ or CD8$^+$ T cells. For two-group comparisons, Mann-Whitney tests were used where appropriate. For comparisons involving multiple treatment conditions, ordinary one-way ANOVA with multiple-comparisons testing was used. ns, not significant; *$p < 0.05$; **$p < 0.01$; ***$p < 0.001$; ****$p < 0.0001$.
See also Figure S5.

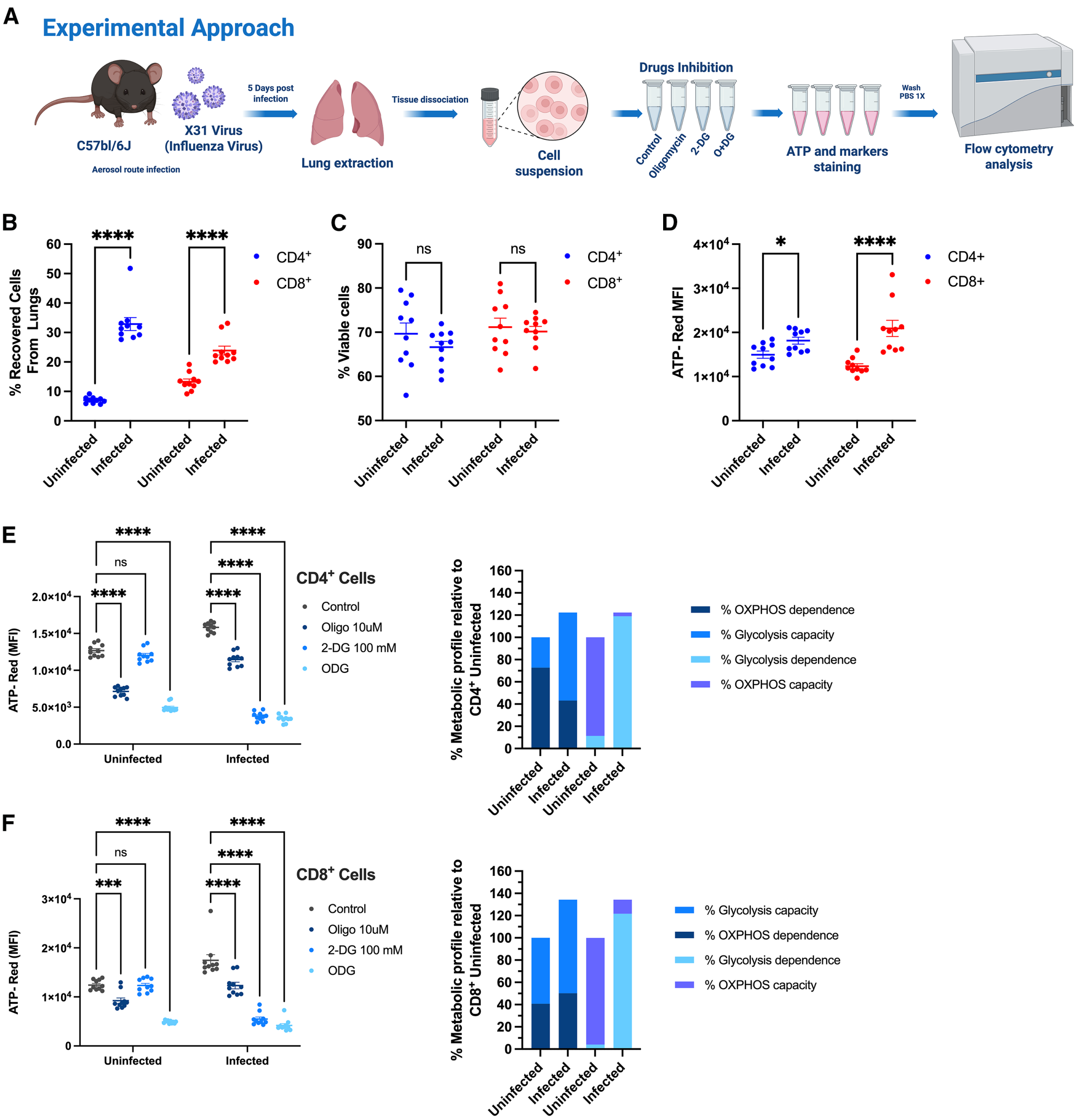

Figure 6. Influenza virus infection alters ATP availability and metabolic profile of CD4⁺ and CD8⁺ T cells in the lung

(A) Schematic of the experimental workflow. C57BL/6J mice were infected with X31 influenza virus. Five days post-infection, lungs were harvested, dissociated, and subjected to metabolic inhibition and ATP-Red staining for flow cytometry analysis.

(B) Quantification of $CD4^+$ and $CD8^+$ T cells recovered from lungs. Infection significantly increased the abundance of both populations.

(C) Viability of recovered $CD4^+$ and $CD8^+$ T cells remained unchanged after infection.

(D) ATP-associated fluorescence measured by ATP-Red in $CD4^+$ and $CD8^+$ T cells increased upon infection.

(E) In $CD4^+$ T cells, inhibitor-based ATP-Red profiling showed a marked reduction in ATP-associated fluorescence under dual inhibition, 2-DG plus oligomycin, in infected cells, consistent with greater glycolytic reliance. Right: inferred metabolic profile showing enhanced glycolytic dependence and altered OXPHOS capacity following infection.

(F) In $CD8^+$ T cells, ATP-associated fluorescence was also reduced under metabolic inhibition, particularly under dual inhibition in infected cells. Right: inferred metabolic profile showing altered pathway balance and reduced relative OXPHOS capacity following infection.

Data are shown as mean ± SEM. Each mouse was considered an independent biological replicate. For (B–D), $n = 10$ mice per group. Technical replicates, when present, were averaged before statistical analysis. ATP-Red fluorescence and viability were quantified by flow cytometry from gated live singlet $CD4^+$ or $CD8^+$ T cells. Statistical analysis was performed using Mann-Whitney tests or ordinary one-way ANOVA with multiple-comparisons testing, as appropriate. ns, not significant; *$p < 0.05$; **$p < 0.01$; ***$p < 0.001$; ****$p < 0.0001$.

See also Figure S6.

Data and code availability

- All data reported in this paper will be shared upon reasonable request.
- This paper does not report original code.
- Any additional information required to reanalyze the data reported in this paper is available upon request.

ACKNOWLEDGMENTS

The authors thank the NHGRI Animal Facility for assistance in generating data for this manuscript. This research was supported in part by the Intramural Research Program of the National Institutes of Health (NIH), including support from the National Human Genome Research Institute (HG200381-13). The contributions of the NIH authors are considered Works of the United States Government. The findings and conclusions presented in this paper are those of the authors and do not necessarily reflect the views of the NIH or the US Department of Health and Human Services.

AUTHOR CONTRIBUTIONS

Conceptualization, J.M. and P.J.M.; methodology, J.M. and P.J.M.; investigation, J.M., B.S., A.L.F., T.T., M.K., S.A., S.M.W., E.M.G.-L., S.K., and P.J.M.; supervision, P.J.M., writing – original draft, J.M. and P.J.M.; writing – review & editing, J.M., B.S., A.L.F., T.T., E.W., S.M.W., E.M.G.-L., A.P.W., and P.J.M.

DECLARATION OF INTERESTS

The authors declare no competing interests.

DECLARATION OF GENERATIVE AI AND AI-ASSISTED TECHNOLOGIES IN THE WRITING PROCESS

During the preparation of this work, the authors used ChatGPT and Grammarly in order to improve the readability and language of the manuscript. After using this tool/service, the authors reviewed and edited the content as needed and take full responsibility for the content of the published article.

STAR★METHODS

Detailed methods are provided in the online version of this paper and include the following:

- KEY RESOURCES TABLE
- EXPERIMENTAL MODEL AND STUDY PARTICIPANT DETAILS
  - Mice
  - Cell lines
- METHOD DETAILS
  - Influenza infection
  - Plasmid transfection
  - Protein synthesis assay
  - SCENITH assay
  - Extracellular flux analysis
  - ATP quantification
  - ATP-red assay
  - Live-cell confocal imaging
  - Image analysis
  - Flow cytometry
  - T cell activation
  - Viability assessment
  - Drugs preparation
- QUANTIFICATION AND STATISTICAL ANALYSIS
  - Statistical analysis

SUPPLEMENTAL INFORMATION

Supplemental information can be found online at https://doi.org/10.1016/j.crmeth.2026.101537.

Received: December 4, 2025
Revised: April 22, 2026
Accepted: July 1, 2026
Published: July 30, 2026

REFERENCES

1. Vardhana, S.A., Hwee, M.A., Berisa, M., Wells, D.K., Yost, K.E., King, B., Smith, M., Herrera, P.S., Chang, H.Y., Satpathy, A.T., et al. (2020). Impaired mitochondrial oxidative phosphorylation limits the self-renewal of T cells exposed to persistent antigen. Nat. Immunol. *21*, 1022–1033. https://doi.org/10.1038/s41590-020-0725-2.
2. Meurer, F., Do, H.T., Sadowski, G., and Held, C. (2017). Standard Gibbs energy of metabolic reactions: II. Glucose-6-phosphatase reaction and ATP hydrolysis. Biophys. Chem. *223*, 30–38. https://doi.org/10.1016/j.bpc.2017.02.005.
3. Horan, M.P., Pichaud, N., and Ballard, J.W.O. (2012). Review: quantifying mitochondrial dysfunction in complex diseases of aging. J. Gerontol. A Biol. Sci. Med. Sci. *67*, 1022–1035. https://doi.org/10.1093/gerona/glr263.
4. Smith, R.L., Soeters, M.R., Wüst, R.C.I., and Houtkooper, R.H. (2018). Metabolic Flexibility as an Adaptation to Energy Resources and Requirements in Health and Disease. Endocr. Rev. *39*, 489–517. https://doi.org/10.1210/er.2017-00211.
5. Ghini, V., Saccenti, E., Tenori, L., Assfalg, M., and Luchinat, C. (2015). Allostasis and Resilience of the Human Individual Metabolic Phenotype. J. Proteome Res. *14*, 2951–2962. https://doi.org/10.1021/acs.jproteome.5b00275.
6. Lane, N., and Martin, W. (2010). The energetics of genome complexity. Nature *467*, 929–934. https://doi.org/10.1038/nature09486.
7. Tantama, M., Martínez-François, J.R., Mongeon, R., and Yellen, G. (2013). Imaging energy status in live cells with a fluorescent biosensor of the intracellular ATP-to-ADP ratio. Nat. Commun. *4*, 2550. https://doi.org/10.1038/ncomms3550.
8. Tantama, M., and Yellen, G. (2014). Imaging changes in the cytosolic ATP-to-ADP ratio. Methods Enzymol. *547*, 355–371. https://doi.org/10.1016/B978-0-12-801415-8.00017-5.
9. Salabei, J.K., Gibb, A.A., and Hill, B.G. (2014). Comprehensive measurement of respiratory activity in permeabilized cells using extracellular flux analysis. Nat. Protoc. *9*, 421–438. https://doi.org/10.1038/nprot.2014.018.
10. Mookerjee, S.A., Goncalves, R.L.S., Gerencser, A.A., Nicholls, D.G., and Brand, M.D. (2015). The contributions of respiration and glycolysis to extracellular acid production. Bba-Bioenergetics *1847*, 171–181. https://doi.org/10.1016/j.bbabio.2014.10.005.
11. Brand, M.D., and Nicholls, D.G. (2011). Assessing mitochondrial dysfunction in cells (vol 435, pg 297, 2011). Biochem. J. *437*, 575. https://doi.org/10.1042/bj4370575u.
12. Argüello, R.J., Combes, A.J., Char, R., Gigan, J.P., Baaziz, A.I., Bousiquot, E., Camosseto, V., Samad, B., Tsui, J., Yan, P., et al. (2020). SCENITH: A Flow Cytometry-Based Method to Functionally Profile Energy Metabolism with Single-Cell Resolution. Cell Metab. *32*, 1063–1075.e7. https://doi.org/10.1016/j.cmet.2020.11.007.
13. Holwerda, A.M., Dirks, M.L., Barbeau, P.A., Goessens, J., Gijsen, A., van Loon, L.J.C., and Holloway, G.P. (2024). Mitochondrial bioenergetics are not associated with myofibrillar protein synthesis rates. J. Cachexia Sarcopenia Muscle *15*, 1811–1822. https://doi.org/10.1002/jcsm.13532.
14. Sasaki, K., Uchiumi, T., Toshima, T., Yagi, M., Do, Y., Hirai, H., Igami, K., Gotoh, K., and Kang, D. (2020). Mitochondrial translation inhibition triggers ATF4 activation, leading to integrated stress response but not to mitochondrial unfolded protein response. Biosci. Rep. *40*, BSR20201289. https://doi.org/10.1042/BSR20201289.
15. Vrieling, F., van der Zande, H.J.P., Naus, B., Smeehuijzen, L., van Heck, J.I.P., Ignacio, B.J., Bonger, K.M., Van den Bossche, J., Kersten, S.,

CellPress
OPEN ACCESS

and Stienstra, R. (2024). CENCAT enables immunometabolic profiling by measuring protein synthesis via bioorthogonal noncanonical amino acid tagging. Cell Rep. Methods *4*, 100883. https://doi.org/10.1016/j.crmeth.2024.100883.

16. Ahl, P.J., Hopkins, R.A., Xiang, W.W., Au, B., Kaliaperumal, N., Fairhurst, A.M., and Connolly, J.E. (2020). Met-Flow, a strategy for single-cell metabolic analysis highlights dynamic changes in immune subpopulations. Commun. Biol. *3*, 305. https://doi.org/10.1038/s42003-020-1027-9.
17. Russo, E., Lemaître, F., Corre, B., Chikina, A.S., Langa-Vives, F., and Bousso, P. (2022). SPICE-Met: profiling and imaging energy metabolism at the single-cell level using a fluorescent reporter mouse. EMBO J. *41*, e111528. https://doi.org/10.15252/embj.2022111528.
18. Wang, L., Yuan, L., Zeng, X., Peng, J., Ni, Y., Er, J.C., Xu, W., Agrawalla, B.K., Su, D., Kim, B., and Chang, Y.T. (2016). A Multisite-Binding Switchable Fluorescent Probe for Monitoring Mitochondrial ATP Level Fluctuation in Live Cells. Angew. Chem. Int. Ed. Engl. *55*, 1773–1776. https://doi.org/10.1002/anie.201510003.
19. Imamura, H., Nhat, K.P.H., Togawa, H., Saito, K., Iino, R., Kato-Yamada, Y., Nagai, T., and Noji, H. (2009). Visualization of ATP levels inside single living cells with fluorescence resonance energy transfer-based genetically encoded indicators. P Natl Acad Sci USA *106*, 15651–15656. https://doi.org/10.1073/pnas.0904764106.
20. Pathak, D., Berthet, A., and Nakamura, K. (2013). Energy failure: does it contribute to neurodegeneration? Ann. Neurol. *74*, 506–516. https://doi.org/10.1002/ana.24014.
21. Kruse, S.E., Watt, W.C., Marcinek, D.J., Kapur, R.P., Schenkman, K.A., and Palmiter, R.D. (2008). Mice with mitochondrial complex I deficiency develop a fatal encephalomyopathy. Cell Metab. *7*, 312–320. https://doi.org/10.1016/j.cmet.2008.02.004.
22. Vafai, S.B., and Mootha, V.K. (2012). Mitochondrial disorders as windows into an ancient organelle. Nature *491*, 374–383. https://doi.org/10.1038/nature11707.
23. Moltedo, B., López, C.B., Pazos, M., Becker, M.I., Hermesh, T., and Moran, T.M. (2009). Cutting edge: stealth influenza virus replication precedes the initiation of adaptive immunity. J. Immunol. *183*, 3569–3573. https://doi.org/10.4049/jimmunol.0900091.
24. Pearce, E.L., Poffenberger, M.C., Chang, C.H., and Jones, R.G. (2013). Fueling immunity: insights into metabolism and lymphocyte function. Science *342*, 1242454. https://doi.org/10.1126/science.1242454.
25. Buck, M.D., Sowell, R.T., Kaech, S.M., and Pearce, E.L. (2017). Metabolic Instruction of Immunity. Cell *169*, 570–586. https://doi.org/10.1016/j.cell.2017.04.004.
26. Picard, M., Prather, A.A., Puterman, E., Cuillerier, A., Coccia, M., Aschbacher, K., Burelle, Y., and Epel, E.S. (2018). A Mitochondrial Health Index Sensitive to Mood and Caregiving Stress. Biol Psychiat *84*, 9–17. https://doi.org/10.1016/j.biopsych.2018.01.012.
27. Pakos-Zebrucka, K., Koryga, I., Mnich, K., Ljujic, M., Samali, A., and Gorman, A.M. (2016). The integrated stress response. EMBO Rep. *17*, 1374–1395. https://doi.org/10.15252/embr.201642195.
28. Murry, C.E., Jennings, R.B., and Reimer, K.A. (1986). Preconditioning with ischemia: a delay of lethal cell injury in ischemic myocardium. Circulation *74*, 1124–1136. https://doi.org/10.1161/01.cir.74.5.1124.

STAR★METHODS

KEY RESOURCES TABLE

REAGENT or RESOURCE	SOURCE	IDENTIFIER
Antibodies		
Alexa Fluor 488 anti-puromycin antibody, clone 2A4	BioLegend	Cat#381505, RRID:AB_2927825
Anti-mouse CD4 FITC antibody, clone GK1.5	Invitrogen	Ref#11-0041-85, RRID: AB_464893
Anti-mouse CD8 APC antibody, clone 53–6.7	BioLegend	Ref#100712, RRID: AB_312751
Anti-mouse CD3ε monoclonal antibody, clone 145-2C11	Bio X Cell	Cat#BE0001-1, RRID: AB_1107634
Anti-mouse CD28 monoclonal antibody, clone PV-1	Bio X Cell	Cat#BE0015-5, RRID: AB_1107628
Bacterial and virus strains		
Mouse-adapted human influenza virus A/X/31, X31	ATCC	VR-1680
Biological samples		
Murine splenocytes from C57BL/6 mice	This paper	N/A
Chemicals, peptides, and recombinant proteins		
Ouabain octahydrate	Sigma-Aldrich	Cat#O3125-250MG
Thapsigargin	Sigma-Aldrich	Cat#T9033-.5MG
2-deoxy-D-glucose	Sigma-Aldrich	Cat#D8375-5G
Oligomycin	Sigma-Aldrich	Cat#405455-10MG
Bongkrekic acid	Cayman Chemical	Cat#38230
Puromycin dihydrochloride	Millipore Sigma	Cat#P8833-25MG
Cycloheximide	Sigma-Aldrich	Cat#C7698
MitoTracker Green FM	Thermo Fisher Scientific	Cat#M7514
CellTracker Deep Red	Thermo Fisher Scientific	Cat#C34565
BioTracker ATP-Red Live Cell Dye	Sigma-Aldrich	Cat#SCT045
Hoechst 33342	Thermo Fisher Scientific	Cat#62249
Trypan blue, 0.04%	Thermo Fisher Scientific	Cat#15250061
0.25% trypsin-EDTA	Gibco	Cat#25200-056
TurboFectin 8.0 Transfection Reagent	OriGene	SKU TF81001
Opti-MEM I Reduced Serum Medium	Thermo Fisher Scientific	Cat#31985070
Critical commercial assays		
Click-iT HPG Alexa Fluor 488 Protein Synthesis Assay Kit	Thermo Fisher Scientific	Cat#C10428
ATP Assay Kit	Sigma-Aldrich	Cat#MAK473
Pierce BCA Protein Assay Kit	Thermo Fisher Scientific	Cat#23228
Fix & Perm Cell Permeabilization Kit	Invitrogen	Cat#GAS001
LIVE/DEAD Fixable Near-IR viability dye	Thermo Fisher Scientific	Cat#L34981
Experimental models: Cell lines		
Dog: MDCK.2 cells	ATCC	CRL-2936, RRID:CVCL_B034
MDCK NDUFS4 knockout cells	iPSC Core, National Heart, Lung, and Blood Institute, National Institutes of Health; Jizhong Zou, Ph.D.	N/A
Experimental models: Organisms/strains		
Mouse: C57BL/6J	The Jackson Laboratory	Strain #000664
Recombinant DNA		
GW1-PercevalHR plasmid	Addgene	Plasmid #49082, RRID:Addgene_21737

(*Continued on next page*)

Continued

REAGENT or RESOURCE	SOURCE	IDENTIFIER
Software and algorithms		
FlowJo v10	BD Biosciences	https://www.flowjo.com
CytExpert	Beckman Coulter	N/A
Fiji/ImageJ	ImageJ	https://imagej.net/software/fiji/
EZColocalization plugin	ImageJ	https://imagej.net/software/fiji/
GraphPad Prism 10	GraphPad Software	Prism 10
ZEN 2.3 blue edition	Zeiss	N/A
Other		
Seahorse XFe96 Analyzer	Agilent	Part#101991-100
CytoFLEX LX cytometer	Beckman Coulter	N/A
Zeiss Yokogawa spinning-disk system	Carl Zeiss	N/A
Lab-Tek II glass-bottom two-chambered slides	Thermo Fisher Scientific	Cat#155379

EXPERIMENTAL MODEL AND STUDY PARTICIPANT DETAILS

Mice

C57BL/6J wild-type mice, also referred to as WT or B6, were purchased from The Jackson Laboratory, strain #000664. Mice were 8–10 weeks old at the time of the experiment, and both male and female mice were used. No specific selection criteria were used for allocation. Mice were assigned to experimental groups based on availability. A total of 10 mice were included in the influenza infected group, consisting of 5 males and 5 females. The uninfected control group was assigned similarly and included 10 mice, consisting of 5 males and 5 females. Male mice weighed 22–28 g, and female mice weighed 17–22 g at the time of the experiment. Mice were group housed, with 5 animals per cage, in a specific pathogen-free facility at 20°C–24°C and 40%–60% humidity under a 12 h light/dark cycle, with *ad libitum* access to standard chow and water. All procedures involving animals were performed in compliance with the Animal Care and Use Committee of the National Human Genome Research Institute under protocol G-11-3.

Cell lines

Madin-Darby canine kidney (MDCK.2) cells (ATCC, CRL-2936) were used in this study. MDCK.2 is an epithelial-like cell line isolated from the distal renal tubule of a normal dog (Canis familiaris). MDCK.2 cells were cultured in Dulbecco's Modified Eagle Medium (DMEM) supplemented with 10% fetal bovine serum and 100 U/mL penicillin-streptomycin at 37°C in a humidified incubator with 5% CO2. Cells were seeded at 1 x 10 ˆ5 or 5 x 10 ˆ4 cells/cm2 and passaged every 72 ± 6 h. Culture flasks, 75 cm2 vent-cap T-flasks, contained 20 mL total medium volume. For passaging, cells were detached using 0.05% trypsin-EDTA (Gibco, Cat#25200-056), neutralized with complete medium, washed with 1x PBS, centrifµged for 5 min, and resuspended in the appropriate medium for downstream assays. Cell concentration and viability were determined by 1:2 dilution in 0.04% trypan blue (Thermo Fisher Scientific, Cat#15250061) using an automated cell counter. Unless otherwise stated, 2 x 10 ˆ5 cells/tube or 1 x 10 ˆ5 cells/well in 96-well plates were used for assays.

NDUFS4-deficient MDCK cells were generated by the iPSC Core at the National Heart, Lung, and Blood Institute, National Institutes of Health, under the direction of Jizhong Zou, Ph.D. The sex of the source animal was not available. Cells tested negative for mycoplasma contamination. No additional cell line authentication was performed beyond obtaining the MDCK.2 cell line directly from ATCC.

METHOD DETAILS

Influenza infection

Mouse-adapted human influenza A/X/31 virus, subtype H3N2, obtained from ATCC (VR-1680), was used for infection. 8 to 10 week old male and female C57BL/6J wild-type mice were exposed to aerosolized 500 TCID50 of X31 in 7 mL saline using a Glas-Col aerosol exposure system. Mice were monitored daily throµghout infection for body weight loss, clinical signs of disease, and survival. Animals were euthanized at 5 days post infection for tissue collection. Euthanasia was performed by CO_2 inhalation followed by cervical dislocation, according to the approved animal protocol.

Plasmid transfection

For ATP/ADP measurements, MDCK cells were transiently transfected with the GW1-PercevalHR plasmid (Addgene, Plasmid #49082) using TurboFectin 8.0 Transfection Reagent (OriGene, SKU TF81001). Twenty-four hours before transfection, cells were plated in complete growth medium to obtain 50%–70% confluence on the day of transfection. For experiments performed in

6-well plates, 1 μg plasmid DNA was diluted in 250 μL Opti-MEM I Reduced Serum Medium (Thermo Fisher Scientific, Cat#31985070) and mixed gently. TurboFectin 8.0 reagent, 3 μL, was added to the diluted DNA, mixed gently, and incubated for 15 min at room temperature to allow complex formation. The transfection mixture was then added dropwise to cells in complete culture medium, and plates were gently rocked to distribute complexes evenly. Cells were incubated at 37°C for 24–48 h and analyzed 24 h after transfection unless otherwise indicated. PercevalHR-transfected cells were subsequently exposed to the metabolic perturbations indicated in the corresponding Results sections, including ATPase inhibition with ouabain and thapsigargin, glycolytic inhibition with 2-deoxy-D-glucose, mitochondrial ATP synthase inhibition with oligomycin, and comparison of wild-type and NDUFS4-deficient cells where indicated. PercevalHR fluorescence was quantified by flow cytometry as an orthogonal live-cell readout of ATP/ADP-related energetic changes.

Protein synthesis assay

Protein synthesis was quantified using the Click-iT HPG Alexa Fluor 488 Protein Synthesis Assay Kit (Thermo Fisher Scientific, Cat#C10428), following the manufacturer's instructions. Unless otherwise indicated, MDCK cells were plated at 1 x 10 ˆ5 cells/well in 96-well plates and exposed to the indicated metabolic inhibitors for 2 h. For HPG labeling, cells were incubated with HPG for 30 min before fixation and click-chemistry detection. Fluorescence was quantified by flow cytometry. In experiments comparing basal translational activity between WT and NDUFS4 knockout cells, cycloheximide was used at 0.5, 1, or 10 μg/mL, and puromycin was used at 10, 100, or 500 μg/mL as translation-inhibition controls where indicated.

SCENITH assay

SCENITH was performed in MDCK WT and NDUFS4 knockout cells to evaluate translational activity as a surrogate of cellular metabolic state. Cells were exposed to metabolic inhibitors at the following final concentrations: vehicle control, 100 mM 2-deoxy-D-glucose, 1 μM oligomycin, or 100 mM 2-deoxy-D-glucose plus 1 μM oligomycin. Cells were incubated with inhibitors for 20–30 min at 37°C and 5% CO2. Without washing, puromycin was then added at a final concentration of 10 μg/mL, and cells were incubated for 15 min at 37°C and 5% CO2. Intracellular puromycin incorporation was used as a measure of protein synthesis and was detected using Alexa Fluor 488-conjμgated anti-puromycin antibody (BioLegend, clone 2A4, Cat#381506). Cell fixation and permeabilization were performed using the Fix & Perm Cell Permeabilization Kit (Invitrogen, Cat#GAS001), according to the manufacturer's instructions. Briefly, after metabolic inhibition and puromycin pulse labeling, cells were harvested, fixed, permeabilized, and stained with anti-puromycin antibody. Fluorescence intensity was quantified by flow cytometry and used to determine relative protein synthesis across conditions.

Extracellular flux analysis

Oxygen consumption rate (OCR) and extracellular acidification rate (ECAR) were measured using a Seahorse XFe96 Analyzer (Agilent, Part#101991-100) in 96-well Seahorse assay plates. MDCK cells were seeded at 4 x 10 ˆ5 cells/well and assayed in XF Assay Modified DMEM under the conditions specified for each experiment. For basal comparisons between WT and NDUFS4-deficient cells, OCR and ECAR were measured under matched assay conditions across genotypes, and values were normalized to protein content.

For mitochondrial stress test assays, Seahorse assay medium was supplemented with 10 mM glucose, 1 mM pyruvate, and 2 mM glutamine. Oligomycin, FCCP, and rotenone/antimycin A were injected at final concentrations of 4 μM, 4 μM, and 1 μM, respectively. For glycolysis stress test assays, Seahorse assay medium was supplemented with 2 mM glutamine. Glucose, oligomycin, and 2-deoxy-D-glucose were injected at final concentrations of 10 mM, 4 μM, and 100 mM, respectively.

For bongkrekic acid experiments, cells were either pre-incubated with BKA for 60 min before extracellular flux analysis or subjected to acute BKA injection during Seahorse acquisition, as indicated in the corresponding Results sections. For pre-incubation studies, mitochondrial respiration and glycolytic parameters were quantified using Mito Stress and Glycolysis Stress assay formats, including basal respiration, ATP-linked respiration, coupling efficiency, and representative OCR/ECAR traces. For acute injection studies, OCR and ECAR were monitored dynamically before and after BKA addition to assess immediate bioenergetic responses. Protein content was quantified using the Pierce BCA Protein Assay Kit (Thermo Fisher Scientific, Cat#23228) according to the manufacturer's instructions. Results are reported as pmol O2/min/μg protein for OCR and mpH/min/μg protein for ECAR, unless otherwise indicated.

ATP quantification

Total intracellular ATP was quantified using the Sigma ATP Assay Kit, Cat#MAK473. MDCK cells, 1 x 10 ˆ6 cells/condition, were treated with the indicated inhibitors, including ouabain 50-100 μM, thapsigargin 50-100 nM, 2-deoxy-D-glucose 50–200 mM, oligomycin 5-20 μM, or combinations thereof, for the time intervals specified in each experiment. Cell lysates were processed according to the manufacturer's instructions, and ATP content was determined by absorbance at 570 nm relative to a standard curve.

ATP-red assay

ATP-associated fluorescence was measured using BioTracker ATP-Red Live Cell Dye, Sigma, Cat#SCT045. MDCK cells were plated at 1 x 10 ˆ5 cells/well in 96-well plates or prepared as 2 x 10 ˆ5 cells/tube unless otherwise indicated. Cells were exposed to the indicated metabolic perturbations for the treatment durations described in each experiment, including 30 min, 2 h, or kinetic

time-course conditions. ATP-Red staining was performed according to the manufacturer's instructions immediately before flow cytometric acquisition. ATP-Red fluorescence was analyzed in gated live singlet cells and expressed relative to the matched control group. Where indicated, ATP-Red was combined with surface marker staining to resolve energetic states within defined immune cell populations.

Live-cell confocal imaging

MDCK cells were seeded on glass-bottom two-chambered slides, Lab-Tek II, Thermo Fisher Scientific, Cat#155379, to approximately 80% confluency. The next day, cells were stained sequentially to label mitochondria, cytoplasm, ATP, and nuclei. Cultures were incubated with 100 nM MitoTracker Green FM, Thermo Fisher, Cat#M7514, and 1 μM CellTracker Deep Red, Thermo Fisher, Cat#C34565, for 30 min at 37°C, followed by 10 min incubation with 2 μM ATP-Red Live Cell Dye, Sigma-Aldrich, Cat#SCT045, and 1 μg/mL Hoechst 33342, Thermo Fisher, Cat#62249. After staining, cells were washed and medium was replaced with phenol-red-free DMEM, Thermo Fisher, Cat#21063029, to minimize background during live imaging.

Live-cell imaging commenced immediately at t = 0 on a Zeiss Yokogawa spinning-disk system mounted on an inverted Zeiss Axio Observer Z1 equipped with a heated stage and environmental chamber maintained at 37°C and 5% CO2. Images were acquired using a Plan-Apochromat 63x/1.4 NA oil-immersion objective and a 16-bit Photometrics Evolve EMCCD camera, 512 x 512 pixels. Typical exposure times were 300–950 ms for DAPI, 90–95 ms for the green channel, 100–120 ms for the red channel, and 300–600 ms for the far-red channel, with EM gain set to 400 for all channels. Laser output percentages at the objective were approximately 50% for 405 nm, 20%–21% for 488 nm, 24%–25% for 561 nm, and 14%–21% for 639 nm. Emission was collected using 450 BP/50, 525 BP/50, 595/50, and 690/50 nm filters, respectively. For each experiment, 24–40 fields of view were recorded using Zeiss Definite Focus, with a time-lapse interval of approximately 1 min 45 s to 2 min 30 s over total durations of 30–45 min depending on the number of positions. Image acquisition and initial processing were performed in ZEN 2.3 blue edition, Zeiss.

Image analysis

Raw image stacks were processed using Fiji/ImageJ software. Co-localization analysis was carried out using the EZColocalization plμgin to calculate Pearson's correlation coefficient between ATP-Red and MitoTracker Green signals, and between ATP-Red and CellTracker Deep Red signals. Quantitative data were further analyzed and visualized using GraphPad Prism. The ratio of Pearson's correlation coefficient values for ATP-Red/MitoTracker Green and ATP-Red/CellTracker Deep Red was calculated.

Flow cytometry

Flow cytometric analyses were performed using a CytoFLEX LX cytometer, Beckman Coulter, equipped with 375, 405, 488, 561, 638, and 808 nm lasers. Cells were stained with anti-mouse CD4 FITC antibody (Invitrogen, clone GK1.5, Ref#11-0041-85) at 7.5 μg/mL per reaction, anti-mouse CD8 APC antibody (BioLegend, clone 53–6.7, Ref#100712) at 3 μg/mL, LIVE/DEAD Fixable Near-IR viability dye (Thermo Fisher, Cat#L34981), and fluorogenic probes for ATP-associated fluorescence and protein synthesis where indicated. Data acquisition was performed using CytExpert, and data analysis was carried out using FlowJo v10. Gating strategies included FSC/SSC to exclude debris, singlet discrimination, and live-cell gating where viability dyes were used, followed by analysis of ATP-Red, PercevalHR, Click-iT HPG, and surface marker signals in the relevant gated single-cell populations. Summary values shown in graphs represent fluorescence intensity measurements derived from these gated single-cell populations, as specified in the corresponding figure legends. Metabolic dependencies were calculated using an SCENITH flow cytometry protocol.

T cell activation

Spleens were harvested from C57BL/6 mice and mechanically dissociated throμgh a 70 μm cell strainer in complete RPMI-1640 medium supplemented with 10% fetal bovine serum, 2 mM L-glutamine, 100 U/mL penicillin, 100 μg/mL streptomycin, and 50 μM beta-mercaptoethanol. Red blood cells were lysed using ACK lysis buffer, and cells were washed twice with PBS. For polyclonal T cell activation, flat-bottom 96-well plates were pre-coated with anti-mouse CD3ε monoclonal antibody (Bio X Cell, clone 145-2C11, Cat#BE0001-1) at 1 μg/mL in sterile PBS and incubated at 4°C overnight. Before cell seeding, plates were washed once with PBS to remove unbound antibody. Splenocytes were plated at 1-2 x 10 ˆ6 cells/well in complete RPMI medium supplemented with soluble anti-mouse CD28 monoclonal antibody (Bio X Cell, clone PV-1, Cat#BE0015-5) at 1 μg/mL. Cells were cultured at 37°C in a humidified incubator with 5% CO2 for 72 h unless otherwise indicated.

Viability assessment

Cell viability was assessed by flow cytometry using LIVE/DEAD Fixable Near-IR viability dye (Thermo Fisher Scientific, Cat#L34981) for 808 nm excitation. Dead cells were excluded from analysis. Viability was quantified relative to untreated controls.

Drugs preparation

Ouabain octahydrate (Sigma-Aldrich, Cat#O3125-250MG), 5 mM stock, and thapsigargin (Sigma-Aldrich, Cat#T9033-.5MG), 1 mM stock, were dissolved in 100% ethanol. 2-deoxy-D-glucose (Sigma-Aldrich, Cat#D8375-5G), 1 M stock, was prepared in Opti-MEM medium. Oligomycin (Sigma-Aldrich, Cat#405455-10MG), 5 mM stock, was prepared in ethanol. Bongkrekic acid (Cayman

Chemical, Cat#38230) was supplied in Tris buffer, pH 7.5, and used in that vehicle for the corresponding experiments. All drμgs were aliquoted and stored at −20°C. Working concentrations were freshly diluted in culture medium before treatment.

QUANTIFICATION AND STATISTICAL ANALYSIS

Statistical analysis

Data are expressed as mean ± SEM from at least three independent experiments unless otherwise indicated. Statistical tests were selected according to the number of experimental groups compared. For comparisons between two groups, Mann-Whitney tests were used. For comparisons involving three or more groups, one-way ANOVA with multiple comparisons was used. A p value <0.05 was considered statistically significant. Statistical analyses and data visualization were performed using GraphPad Prism.

Cell Reports Methods, Volume *6*

Supplemental information

ATP-Red enables single-cell bioenergetic phenotyping by flow cytometry

Jose Marin, Bharati Singh, Amanda Fuchs, Tatiana Tarasenko, Emily Warren, Martha Kirby, Stacie Anderson, Stephen M. Wincovitch, Eliza M. Gordon-Lipkin, Shannon Kruk, A. Phillip West, and Peter J. McGuire

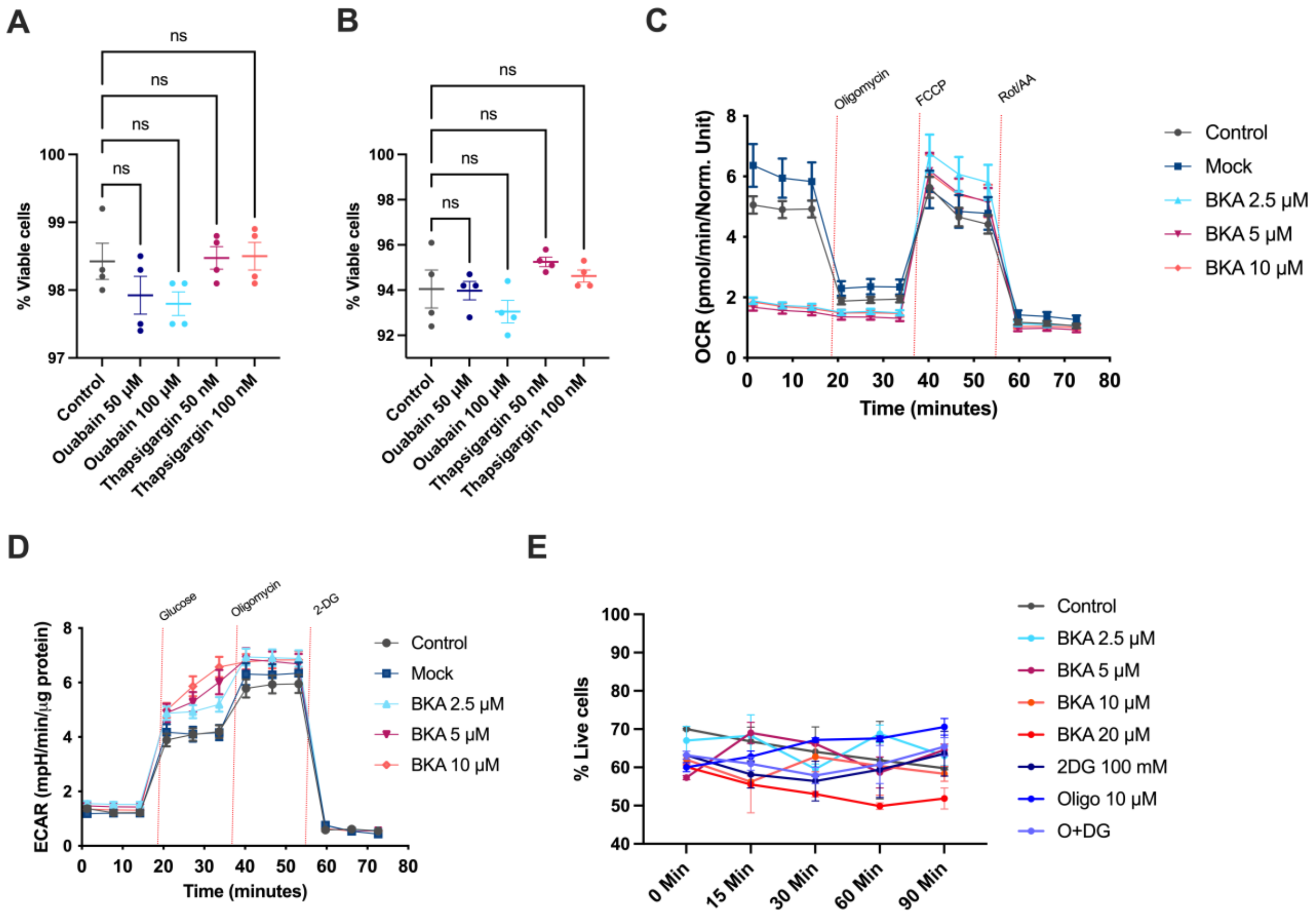

Figure S1. Cell viability and acute extracellular flux responses associated with ATPase inhibition, ANT inhibition, and short-term metabolic perturbation in MDCK cells. (A-B) Cell viability in MDCK cells after treatment with ouabain or thapsigargin under the conditions used for ATP accumulation experiments. Viability remained high across all conditions, with no significant reduction relative to control. (C) Representative OCR traces from Seahorse Mito Stress analysis of control and BKA-treated MDCK cells following sequential injection of oligomycin, FCCP, and rotenone/antimycin A. (D) Representative ECAR traces from Seahorse Glycolysis Stress analysis of control and BKA-treated MDCK cells following sequential injection of glucose, oligomycin, and 2-DG. (E) Cell viability during short term kinetic ATP-Red experiments in MDCK cells treated with BKA, 2-DG, oligomycin, or oligomycin plus 2-DG for the indicated times. Viability remained broadly stable during the 90 min acquisition period across all treatment conditions. Data are shown as mean ± SD. Statistical analysis for panels A-B was performed using ordinary one-way ANOVA followed by multiple-comparisons testing against control. Panel F was analyzed by ordinary two-way ANOVA with treatment and time as factors, followed by multiple-comparisons testing as indicated. Panels D-E are representative Seahorse traces. ns, not significant; *P < 0.05; **P < 0.01; ***P < 0.001; ****P < 0.0001.

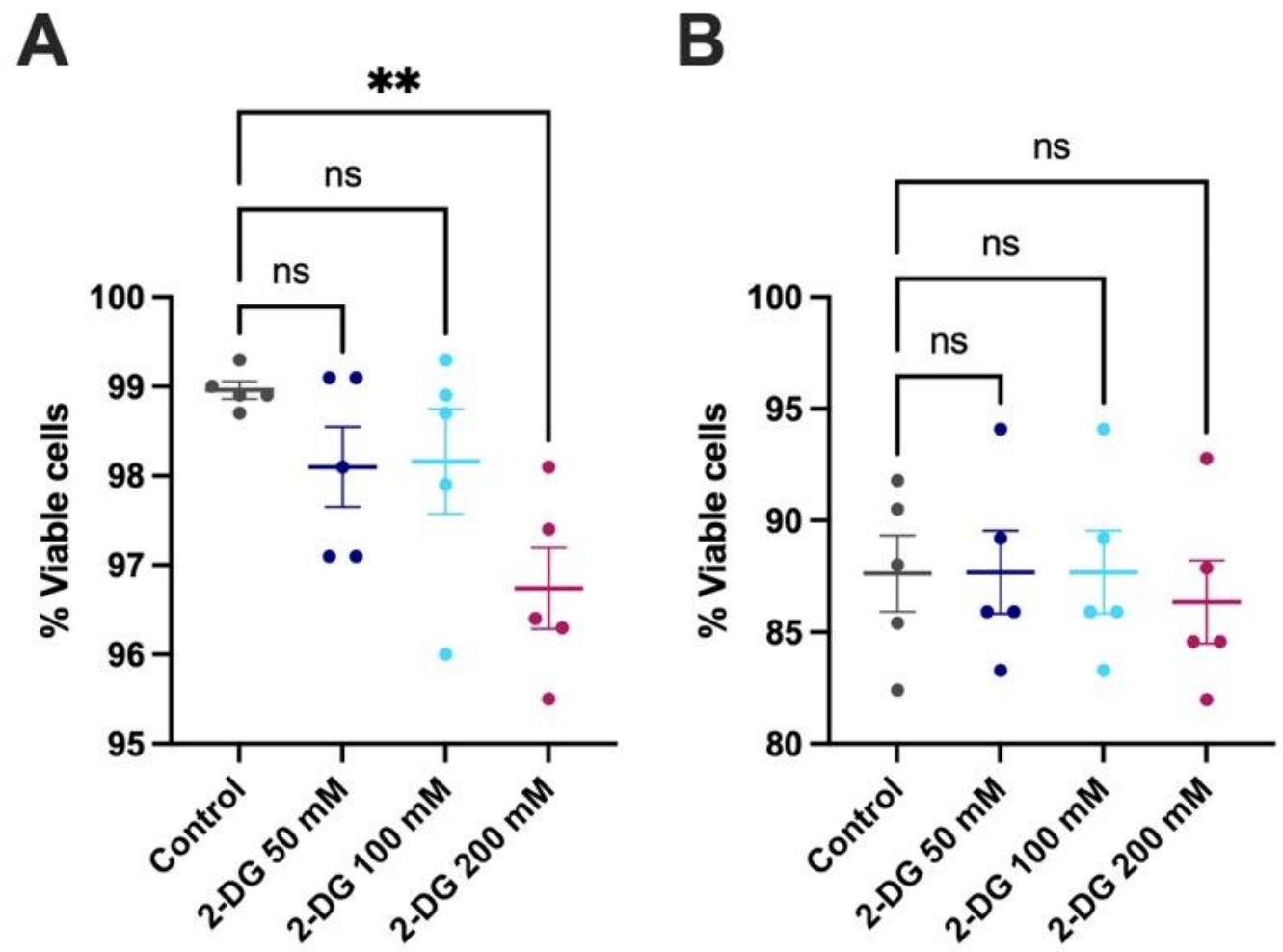

Figure S2. Cell viability remains preserved during glycolytic inhibition by 2-deoxy-D-glucose in MDCK cells. (A-B) Cell viability in MDCK cells treated with increasing concentrations of 2-deoxy-D-glucose (2-DG) under the conditions used for ATP/ADP, ATP-Red measurements. Viability remained high across all treatment conditions, with only modest changes at the highest dose. Data are shown as mean ± SD. Statistical analysis was performed using ordinary one-way ANOVA followed by multiple-comparisons testing against control. ns, not significant; *$P < 0.05$; **$P < 0.01$; ***$P < 0.001$; ****$P < 0.0001$.

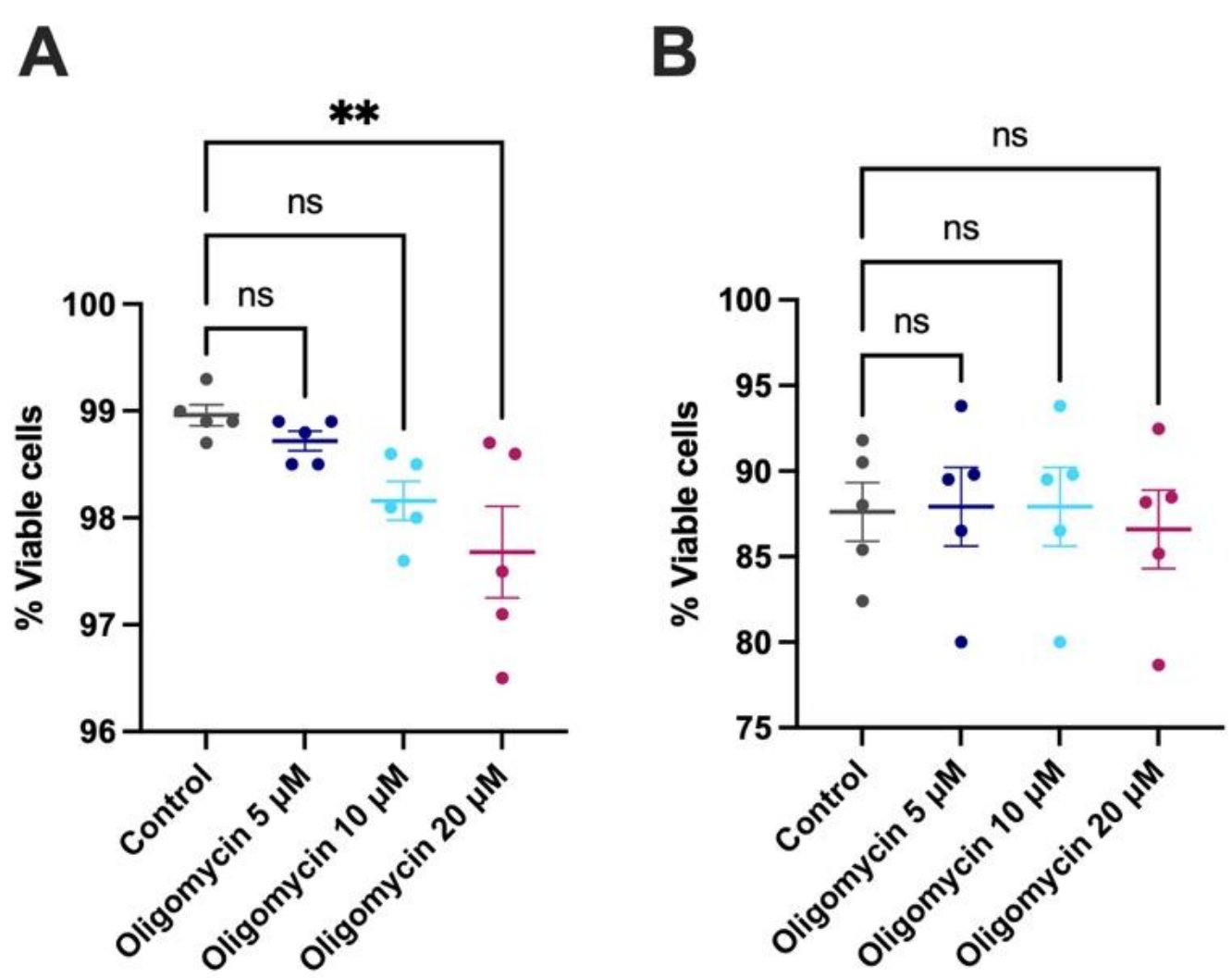

Figure S3. Cell viability remains largely preserved during mitochondrial ATP synthase inhibition by oligomycin in MDCK cells. (A-B) Cell viability in MDCK cells treated with increasing concentrations of oligomycin under the conditions used for ATP/ADP, ATP-Red measurements. Viability remained high across all treatment groups, with only limited reduction at the highest concentration. Data are shown as mean ± SD. Statistical analysis was performed using

ordinary one-way ANOVA followed by multiple-comparisons testing against control. ns, not significant; *P < 0.05; **P < 0.01; ***P < 0.001; ****P < 0.0001.

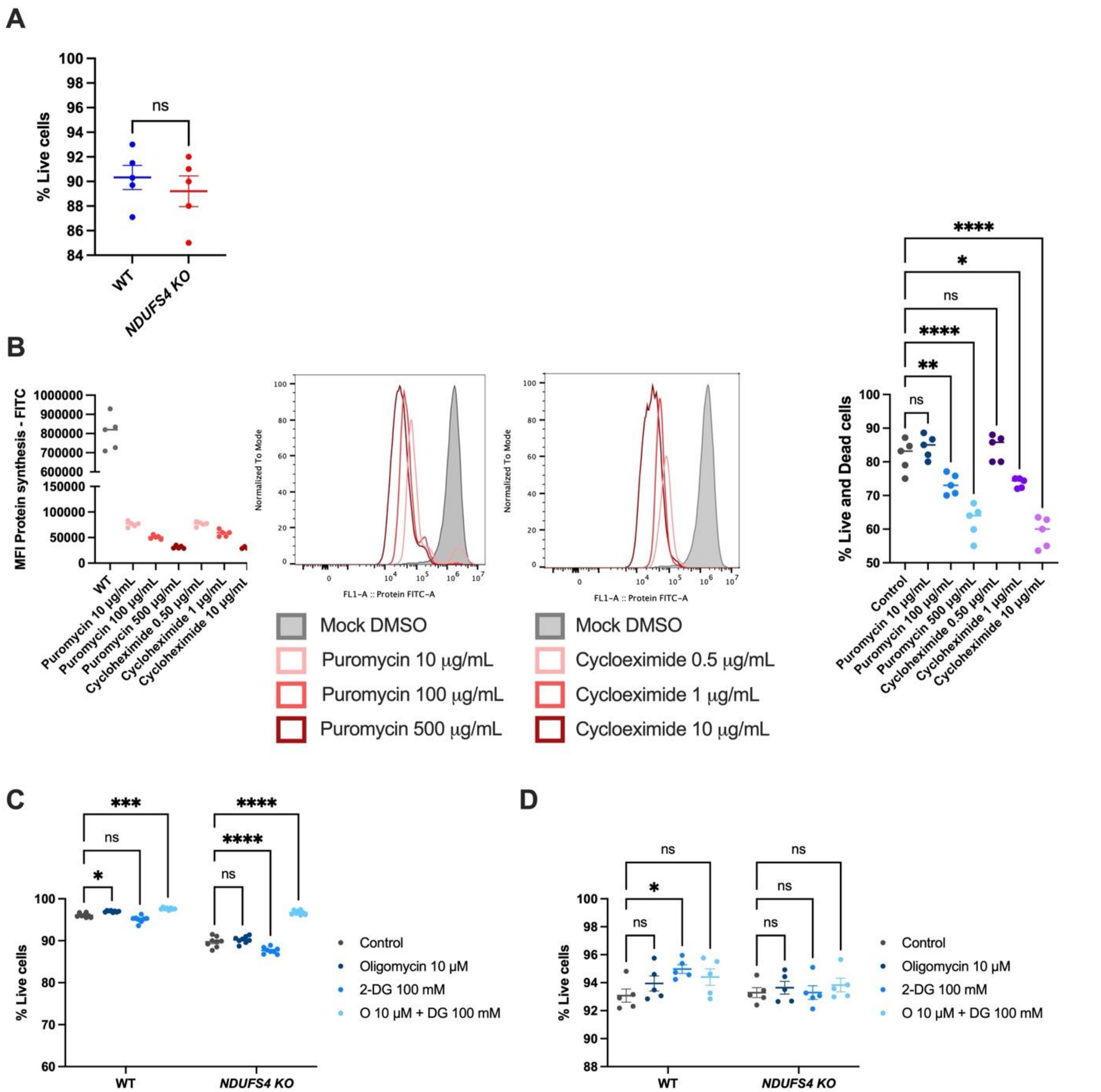

Figure S4. Viability controls and puromycin optimization for metabolic phenotyping in WT and *NDUFS4 KO* MDCK cells. (A) Baseline viability of WT and NDUFS4 KO MDCK cells. No significant difference in viability was observed between genotypes under untreated conditions. (B) Optimization of puromycin and cycloheximide treatment conditions for protein synthesis measurements in WT MDCK cells. Left, mean fluorescence intensity of protein synthesis signal across puromycin and cycloheximide conditions. Middle and right, representative histograms illustrating progressive reduction in protein synthesis signal with increasing puromycin or cycloheximide treatment relative to mock control. Far right, percentage of live and dead cells

across the indicated puromycin and cycloheximide conditions. (C-D) Viability of WT and *NDUFS4 KO* MDCK cells after treatment with oligomycin, 2-DG, or oligomycin plus 2-DG under the conditions used for ATP-Red-based metabolic dependence analysis. Viability remained broadly preserved across conditions, with only modest treatment-associated changes. Data are shown as mean ± SD. Statistical analysis for panel A was performed using unpaired two-tailed Student's t test. Panels B–D were analyzed using ordinary one-way ANOVA or two-way ANOVA, as appropriate for grouped comparisons, followed by multiple-comparisons testing as indicated on the graphs. Representative histograms are shown in panel B. ns, not significant; *P < 0.05; **P < 0.01; ***P < 0.001; ****P < 0.0001.

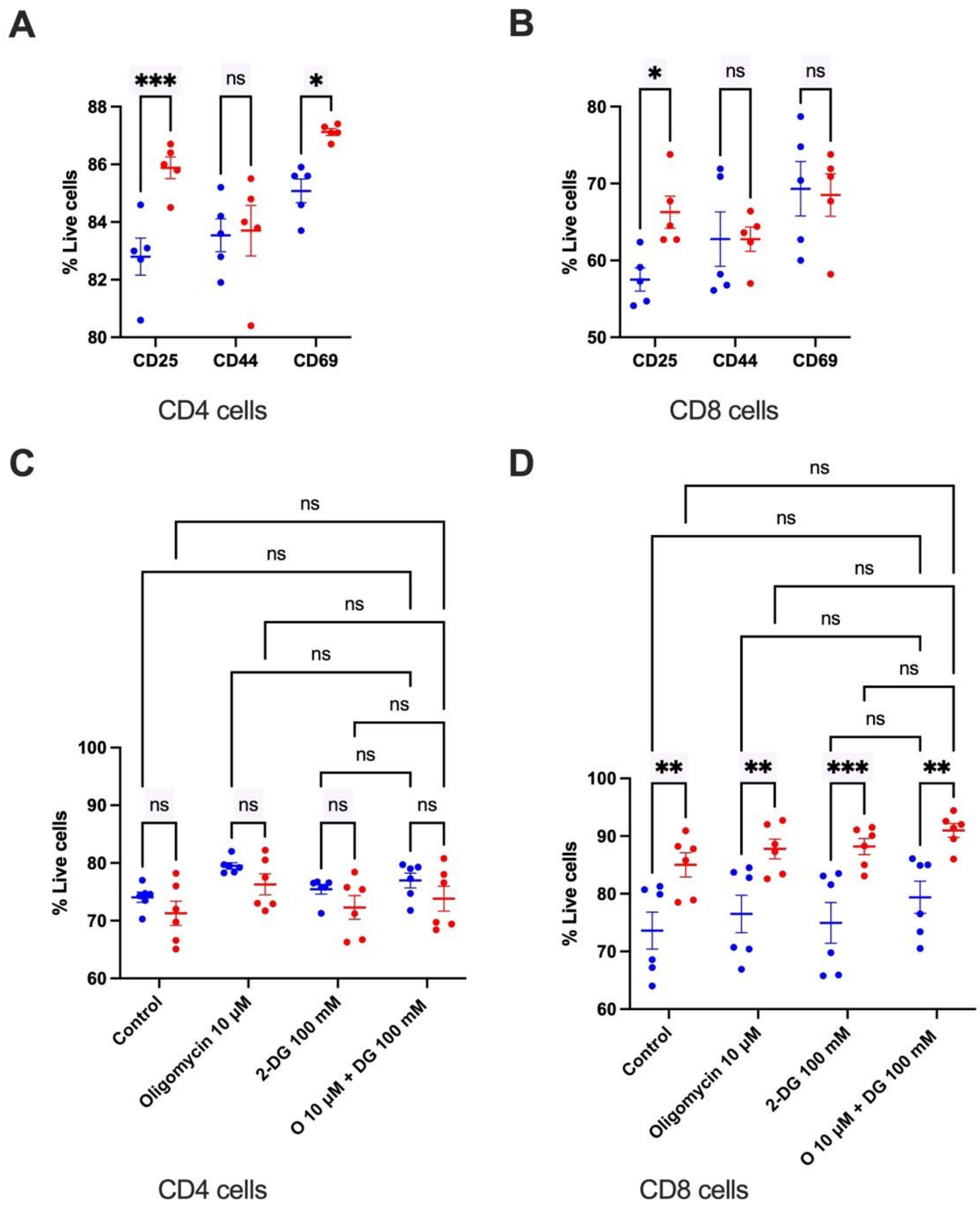

Figure S5. Viability controls for ATP-Red-based metabolic profiling during T cell activation. (A-B) Viability of activated-marker-positive CD4+ and CD8+ T cells after anti-CD3/CD28 stimulation. Activated cells expressing CD25, CD44, or CD69 maintained equal or greater viability than their resting counterparts. (C-D) Viability of CD4+ and CD8+ T cells under control conditions or after treatment with oligomycin, 2-DG, or oligomycin plus 2-DG during ATP-Red-based metabolic dependence analysis. Viability remained broadly preserved across all treatment groups, including under dual inhibition, with CD8+ T cells showing robust survival. Data are shown as mean ± SD. Statistical analysis was performed using ordinary two-way ANOVA followed by multiple-comparisons testing as indicated. ns, not significant; *P < 0.05; **P < 0.01; ***P < 0.001; ****P < 0.0001.

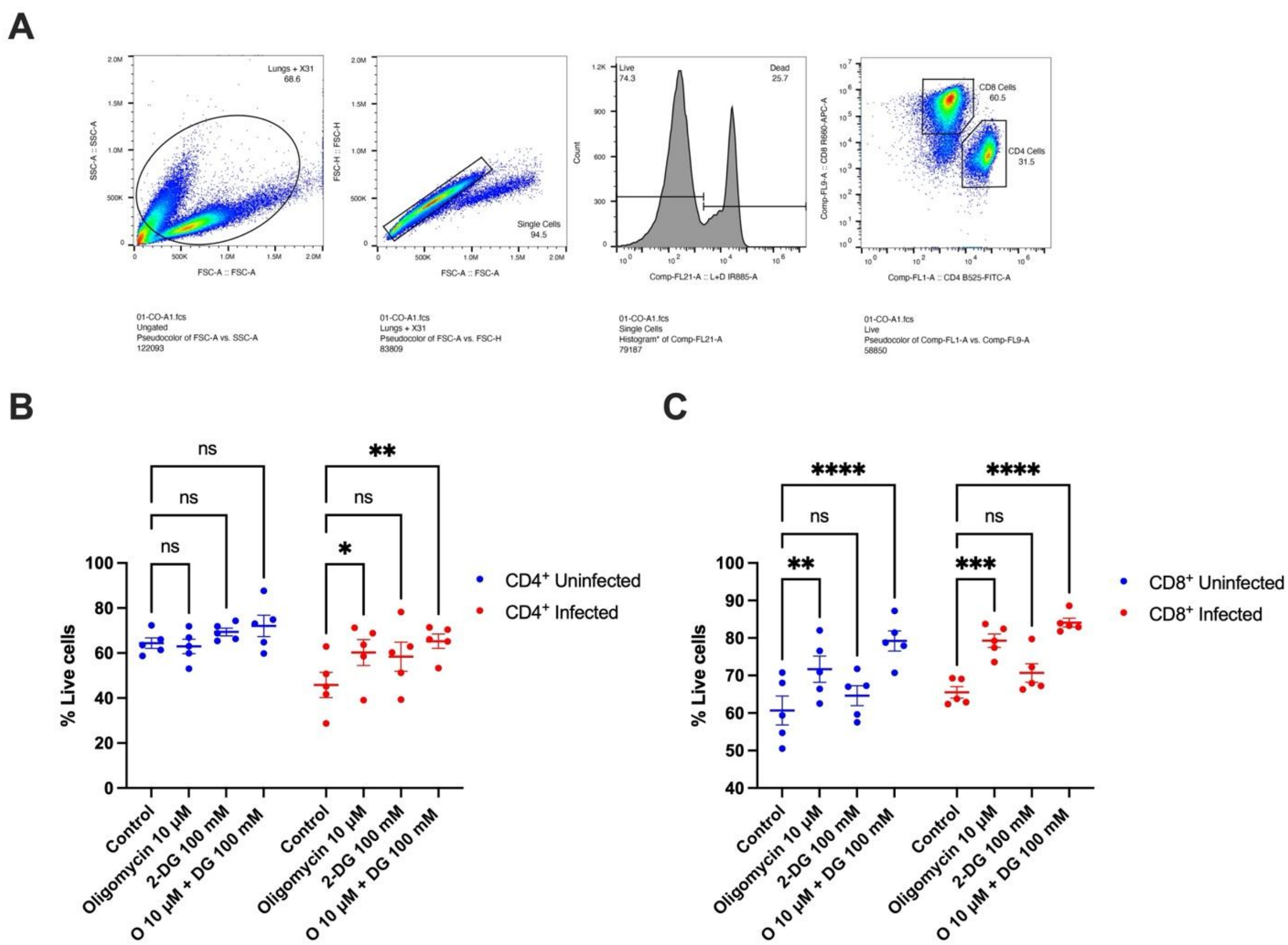

Figure S6. Gating strategy and viability controls for ATP-Red-based metabolic profiling of lung T cells from influenza-infected mice. (A) Representative flow cytometry gating strategy for ex vivo lung T cell analysis from influenza X31 infected mice. Sequential gates show selection of leukocyte-enriched events by FSC/SSC, singlet discrimination, live-cell gating using viability dye exclusion, and final identification of CD4+ and CD8+ T cell populations. (B) Viability of CD4+ and CD8+ T cells isolated from lungs of uninfected or influenza-infected mice after treatment with oligomycin, 2-DG, or oligomycin plus 2-DG under the conditions used for ATP-Red-based

metabolic dependence analysis. Baseline viability was maintained, but infected-lung T cells showed increased sensitivity to dual metabolic inhibition relative to uninfected controls. Data are shown as mean ± SD. Statistical analysis for panel B was performed using ordinary two-way ANOVA followed by multiple-comparisons testing as indicated. Panel A shows representative gating plots. ns, not significant; *$P < 0.05$; **$P < 0.01$; ***$P < 0.001$; ****$P < 0.0001$.
